# Use of acupuncture in stroke and stroke complications: a systematic review and meta-analysis based on sham-controlled trials

**DOI:** 10.3389/fneur.2025.1668497

**Published:** 2025-10-22

**Authors:** Xiaoyu Wang, Jinquan Li, Kuan Yao, Zhiwen Cheng, Tao Jiang, Wei Liu, Huan Li, Bowen Xiong, Hao Zhu, Xiaofei Zhang, Wenjing Song, Yuxin Lu

**Affiliations:** ^1^The First Clinical Medical College, Anhui University of Chinese Medicine, Hefei City, Anhui Province, China; ^2^Massage Department, The Second Affiliated Hospital of Anhui University of Traditional Chinese Medicine, Hefei City, Anhui Province, China; ^3^The Second Clinical Medical College, Anhui University of Chinese Medicine, Hefei City, Anhui Province, China; ^4^Acupuncture Department, First Teaching Hospital of Tianjin University of Traditional Chinese Medicine, Tianjin City, China; ^5^National Clinical Research Center for Chinese Medicine Acupuncture and Moxibustion, Tianjin City, China; ^6^Center for Characteristic Therapies of Traditional Chinese Medicine, The First Affiliated Hospital of Henan University of Chinese Medicine, Zhengzhou City, Henan Province, China

**Keywords:** stroke, stroke complications, acupuncture, blind design, sham acupuncture

## Abstract

**Objective:**

To evaluate the efficacy of acupuncture in sham-controlled trials for stroke and its complications, compare the clinical differences between acupuncture groups and sham groups, and assess potential factors contributing to these differences.

**Methods:**

Medical Subject Headings searches of 7 databases were conducted from January 1, 2000, to December 31, 2024. The primary outcome was the Barthel Index (BI), and the secondary outcomes included the scores of the National Institute of Health Stroke Scale (NIHSS), Quality of Life Scale (QOL), Hamilton Depression Scale (HAMD), and adverse events. Meta-analysis systematically compared acupuncture with sham/placebo acupuncture, analyzing pooled data according to distinct quantitative acupuncture factors, with their corresponding 95% confidence intervals (CIs). Random-effects modeling was performed to pool the effect sizes. The quality of RCTs and evidence was evaluated by the Risk of Bias Tool and the Grading of Recommendations Assessment, Development, and Evaluation approach (GRADE), respectively.

**Results:**

A total of 24 RCTs were included. The risk of bias was usually with some concerns. Compared with sham/placebo acupuncture, acupuncture significantly improved neurological function as measured by the NIHSS [7trails, *n* = 963; mean difference (MD) −1.10, 95%CI −1.94 to −0.26; GRADE low], enhanced quality of life assessed by the Stroke Specific Quality of Life scale(SSQOL) score (3trails, *n* = 756; MD 13.91, 95%CI 6.02 to 21.80; GRADE moderate), and reduced depressive symptoms according to the HAMD score(5trails, *n* = 361; SMD −0.54, 95%CI −1.11 to −0.03; GRADE low). However, there was no difference in the daily living ability measured by BI score (5trails, *n* = 454; MD 2.58, 95%CI 0.51 to 4.66; GRADE low). The variations in needling depth, type, manual manipulation, and de qi, could lead to significant differential effects.

**Conclusion:**

These findings suggest that acupuncture is associated with improved life quality, neurological function, and depressive symptoms in stroke patients, although it does not appear to enhance abilities of daily living. Future research should ascertain whether needling depth, type, manual manipulation, and de qi are correlated with optimal acupuncture strategies and sham-control design methodologies.

**Systematic review registration:**

https://www.crd.york.ac.uk/prospero/, identifier CRD42023378930.

## 1 Introduction

Stroke represents a widespread public health concern. Globally, it ranks as the second leading cause of death and a primary contributor to disability [5.7% (5.1–6.2) of total disability-adjusted life-years, DALYs] (1). Over the past three decades, the absolute number of stroke patients has escalated by 70.0%, with recent evidence indicating a shift towards younger age groups (2, 3). As the population of stroke survivors grows, post-stroke rehabilitation services and the global burden of disability face persistent challenges (4). Enhancing effective rehabilitation interventions and alleviating the medical burden of stroke are priorities in global healthcare (5).

Acupuncture, recognized as a complementary and alternative therapy, has gained widespread international application. Currently, the mechanisms underlying the effects of acupuncture in treating stroke and its complications remain elusive. Potential explanations suggest that acupuncture may protect the central nervous system and cerebrovascular function by maintaining the integrity of the blood–brain barrier (BBB), reducing neuronal apoptosis, and stimulating neuronal reorganization (6–8). Furthermore, acupuncture promotes the recovery of neurological, motor, and cognitive functions (9, 10) by activating various physiological pathways in both the central and peripheral nervous systems (11, 12). Both the World Health Organization (WHO) and the National Institutes of Health (NIH) have included acupuncture as a therapeutic approach for stroke and its complications (13, 14).

Although the number of RCTs evaluating the efficacy of acupuncture for stroke and its complications has been increasing, its effectiveness remains controversial. The varying quality of studies and methodological flaws have made it challenging to evaluate the effectiveness of acupuncture objectively (15, 16). A necessary condition for generating high-quality evidence is to continuously optimize the trial design using practical recommendations, ensuring that the treatment methods meet the needs of clinical practice. As a technical intervention, acupuncture is characterized by personalized treatment plans, significant technical operability, and doctor-patient interaction. This poses requirements for the standardization, reproducibility, blinding, and sham acupuncture design of acupuncture RCTs (17). Given the complexity and specificity of acupuncture, there is much to learn from RCTs employing blinding and sham/placebo acupuncture designs over the past two decades.

This study summarizes the existing evidence on acupuncture treatment for stroke and its complications, utilizing blinded and sham acupuncture designs through a systematic review and meta-analysis. The specific research questions are as follows: (1) the relationship between acupuncture treatment and the improvement of stroke-related dysfunction; (2) the difference in therapeutic effects between sham/placebo acupuncture and real acupuncture treatment; (3) a description of the current application status of sham/placebo acupuncture controls, and a discussion on the main factors causing clinical differences due to different control methods.

## 2 Methods

This systematic review and meta-analysis adhered to the Preferred Reporting Items for Systematic Reviews and Meta-Analyses (PRISMA) reporting guideline, with its peer-reviewed protocol published online (18–20) (PROSPERO registration No. CRD42023378930).

### 2.1 Literature search

Searches were performed in 7 electronic databases: PubMed, Cochrane Library, Embase, Web of Science, China National Knowledge Infrastructure (CNKI), Chongqing VIP (CQVIP), and WanFang Data (all searched from January 1, 2000 to December 31, 2024). Briefly, the following search terms were used: a combination of free words and Medical Subject Headings (MeSH), including stroke, cerebral infarction, cerebral hemorrhage, acupuncture, sham acupuncture, placebo acupuncture, and non-acupoint acupuncture. The search terms are listed in Supplementary Appendix 1. After excluding duplicates, two of us (XYW and JQL) independently screened titles and abstracts and reviewed the full text for each potential inclusion. Review authors (KY) resolved discrepancies to achieve consensus.

### 2.2 Study selection and study eligibility

We included RCTs that involved patients with stroke without limitations on age, sex, type, degree, and duration of stroke. The intervention was acupuncture therapy compared with a sham/placebo acupuncture control design. Besides acupuncture and electroacupuncture, cupping, moxibustion, bloodletting therapy, acupoint injection, application, and catgut embedding therapy are all excluded from the intervention group (IG). The sham acupuncture (e.g., non-acupoints, minimal manipulation, or shallow puncture under the skin) was set as a type of penetrating sham acupuncture. Non-penetrating blunt and retractable needles are considered placebo acupuncture. Studies involving double-blind and double-simulated design were also included.

The primary outcome of interest was the daily living ability assessed by the BI, the activities of daily living (ADL), or the modified Barthel Index (MBI). The secondary outcome was the score of neurological defection [e.g., the NIHSS, the modified Rankin Scale (mRS), and the Chinese Stroke Scale (CSS)], life quality [e.g., the World Health Organization Quality of Life (QOL), the SSQOL], and all indicators which can evaluate stroke-related complications (e.g., limb function, depression, dysphagia, or sleep disorders).

Indicators appearing more than three times were incorporated into the meta-analysis. Specifically, in the included studies: (1) if multiple indicators were used within the same category in one study, we followed the order of preference as described in the example above; (2) if the same indicator was measured multiple times, we prioritized the results obtained at the end of treatment, as this could be the time point reflecting the maximum benefit of the acupuncture intervention being evaluated; and (3) only when the total number of times of reported indicators at the end of treatment was less than three, we considered incorporating results from other observation time points into the analysis.

To enhance the representativeness of the included studies, we excluded those with unclear diagnoses, single-group sample sizes of less than 15, missing data, incorrect data expressions, or studies without accessible full texts. For articles stemming from the same research project, we opted for the most recent publication date.

### 2.3 Data extraction and quality assessment

For included studies, basic characteristics, first author, publication year, country, sample size, age, and clinical variables (including type of stroke, treatment methods, and clinical outcomes) were extracted using a pre-piloted Excel form. If the data were missing, the respective authors of individual studies were contacted by email.

The quality assessment and risk of bias within individual RCTs was conducted by two independent authors (JQL and KY). Certainty of evidence rating for each outcome was carried out using the Grades of Recommendations, Assessment, Development, and Evaluation (GRADE) approach (21). Overall details of acupuncture treatment and risk of bias for each study were assessed using the Standards for Reporting Interventions in Controlled Trials of Acupuncture (STRICTA) 2010 checklist (22) and The Risk of Bias Tool (ROB 2.0), respectively. Any discrepancies and disagreements were resolved through discussion with a panel of other authors.

### 2.4 Statistical analysis

The primary characteristics of including studies were qualitatively pooled. All statistical analyses and plotting were conducted using Review Manager, version 5.4 (Cochrane), and RStudio, version 4.2.1. The I2 statistic was employed to assess statistical heterogeneity. Significant statistical heterogeneity was defined as I2 greater than 50% and *p* < 0.10. We accounted for the subgroup variations (acupuncture vs. sham/placebo acupuncture control) and pooled these in a random-effects model to assess potential sources of heterogeneity. The effect estimates were expressed using the mean difference (MD) or the standardized mean difference (SMD) with 95%CIs. To assess publication bias, we performed Begg’s and Egger’s tests, examining funnel plots for asymmetry when the number of included studies was adequate (10 or more).

## 3 Results

### 3.1 Study selection and characteristics

**Figure 1:**
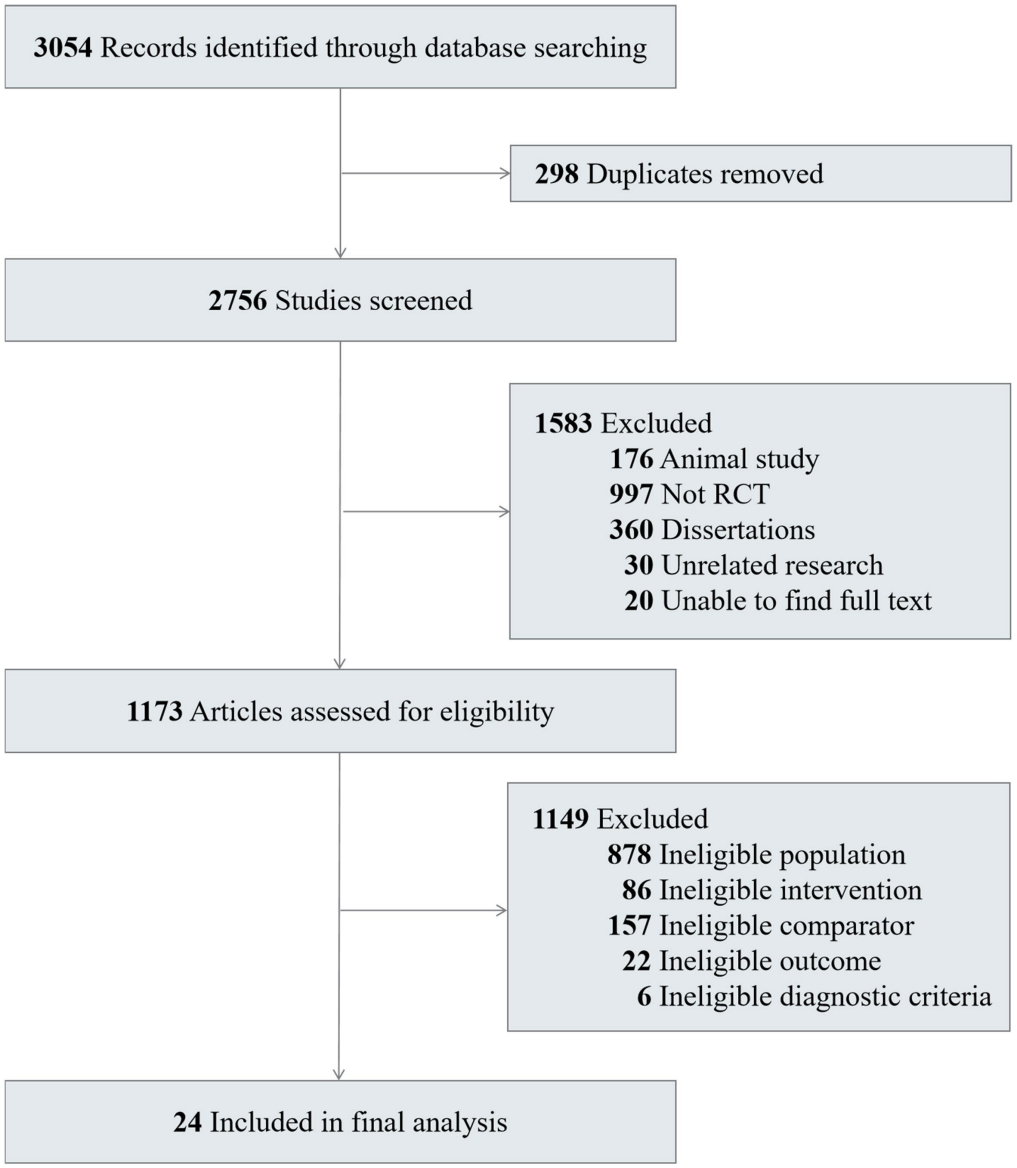
Flow diagram of screened and selected studies.

A total of 3,054 records were identified, and 24 RCTs involving 2,310 participants were included in this review (Figure 1) (18–20, 23–45). The sample size varied from 37 to 287 in the included RCTs, with 1,168(50.56%) participants in the experimental group and 1,142 (49.44%) in the control group. The study characteristics contributing to each trial are presented in Table 1 and Supplementary Table 1. All studies were conducted in China and Korea. There was significant variation in the types of stroke complications. The included 24 studies discussed 8 complications after stroke: depression (6 studies), dysphagia (3 studies), limb dysfunction (3 studies), insomnia (3 studies), aphasia (2 studies), cognitive impairment (2 studies), neurological dysfunction (1 study), and anxiety disorder (1 study). The results that meet the selection criteria of this study including the score of BI, NIHSS, SSQOL and HAMD. The patient inclusion criteria in 11 studies restricted disease course within 3 months. A majority of the studies included did not establish follow-up points, with only 3 studies reporting clinical outcomes beyond 6 months of follow-up.

**Table 1:** Summary of the included trials.

Sources	Country	Type of stroke (complications)	Stroke course	Patients, No.		Treatment		Adverse events, No (%)		Risk of bias
				IG	CG	IG	CG	IG	CG	
Shen et al. (26)	China	CI (NA)	< 2wks	144	143	AT	SAT	12 (8.33%)	22 (15.38%)	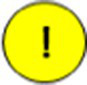
Xia et al. (30)	China	Stroke (DAS)	< 3mo	61	55	AT	SAT	5 (8.20%)	3 (5.45%)	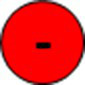
Li et al. (28)	China	Stroke (PSLD)	< 3wks	121	117	AT	SAT	NR	NR	*** 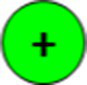
You et al. (33)	China	Stroke (PSD)	NR	34	33	AT	SAT	2 (5.88%)	0 (0.00%)	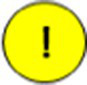
Li et al. (24)	China	Stroke (PSD)	>3mo	23	20	AT, PP	SAT, Drug	3 (13.04%)	3 (15.00%)	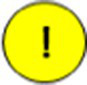
Lee et al. (23)	Korea	Stroke (PSI)	NR	27	25	AT	SAT	NR	NR	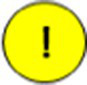
Zheng et al. (25)	China	Stroke (PSA)	NR	41	37	AT	SAT	NR	NR	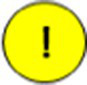
Liao et al. (31)	China	Stroke (NA)	< 2wks	28	20	AT	SAT	NR	NR	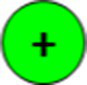
Tsai et al. (44)	China	CI (NA)	< 1 day	18	19	AT	SAT	2 (11.11%)	0 (0.00%)	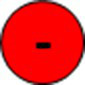
Li et al. (37)	China	ICH (NA)	< 1wks	35	37	AT	SAT	21 (60.00%)	1 (2.70%)	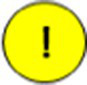
Li et al. (41)	China	CI (PSA)	15–90 days	115	116	AT	SAT	3 (2.61%)	3 (2.59%)	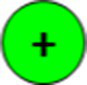
Liu et al. (27)	China	Stroke (NA)	< 2wks	33	33	Eacu	Sham Eacu	NR	NR	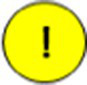
Ma et al. (43)	China	CI (PSD)	3mo–1y	72	71	Eacu, PP	Sham Eacu, Drug	4 (5.56%)	3 (4.23%)	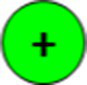
Wang et al. (19)	China	Stroke (ND, PSLD)	2wks–3mo	30	29	AT	SAT	NR	NR	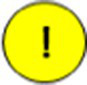
Liu et al. (34)	China	Stroke (PSD)	< 1y	30	30	AT, PP	SAT, Drug	2 (6.67%)	6 (20.00%)	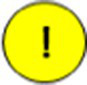
Xiong et al. (32)	China	Stroke (PSCI, PSLD)	> 3wks	35	35	AT	SAT	NR	NR	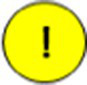
Qian et al. (29)	China	Stroke (PSD)	<6mo	32	33	AT, PP	SAT, Drug	5 (14.71%)	1 (2.94%)	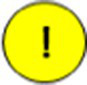
Li et al. (39)	China	Stroke (PSCI)	2wks–6mo	34	34	AT	SAT	1 (2.94%)	1 (2.94%)	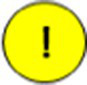
Li et al. (38)	China	CI (DAS)	15 days–6mo	30	30	Eacu	Sham Eacu	NR	NR	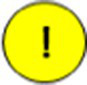
Zheng et al. (40)	China	CI (DAS)	2–6wks	60	60	Eacu	Sham Eacu	NR	NR	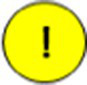
Li et al. (42)	China	Stroke (PSAD)	2wks–3mo	26	28	AT	SAT	2 (7.14%)	0 (0.00%)	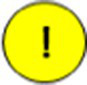
Cao et al. (36)	China	CI (PSI)	2wks–2y	72	72	AT	SAT	2 (2.78%)	1 (1.39%)	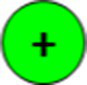
Cai et al. (35)	China	CI (PSD)	NR	33	32	Eacu	Sham Eacu	2 (6.06%)	0 (0.00%)	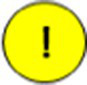
Zhang et al. (45)	China	CI (PSI)	3–12mo	34	33	Eacu	Sham Eacu	NR	NR	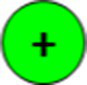

CI, Cerebral Infarction; CG, Control Group; DAS, Dysphagia After Stroke; ICH, Intracerebral Hemorrhage; IG, Intervention Group; NA, Not Applicable; ND, Neurological Dysfunction; NR, Not Reported; PSA, Post-stroke Aphasia; PSAD, Post-stroke Anxiety Disorder; PSCI, Post-stroke Cognitive Impairment; PSD, Post-stroke Depression; PSI, Post-stroke Insomnia; PSLD, Post-stroke Limb Dysfunction; 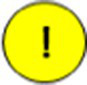, High risk; 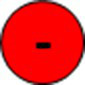, Some concerns; 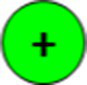, Low risk.

Sham acupuncture details of the control group, including treatment type, acupoint location, acupoint quantity, needling instrument, acupoint depth, manual manipulation, de qi, electroacupuncture, and retention time, are summarized in Table 2 and Supplementary Table 2. Various studies employed different comparison strategies for the intervention and control groups: 4 (16.67%) trials compared acupuncture and placebo with sham acupuncture and drugs (24, 29, 34, 43); 6 (25%) trials compared electroacupuncture with sham electroacupuncture (27, 35, 38, 40, 43, 45); and 15 (62.5%) trials compared acupuncture with sham acupuncture. The most prevalent methods in sham acupuncture control designs are to select non-acupoint locations adjacent to the actual acupoints, utilize conventional needles, not puncture, without employing any manipulation techniques, and no sensation of “deqi” is experienced.

**Table 2:** Characteristic of sham/placebo acupuncture controls application status of 24 primary publications included in the analysis.

Characteristic	Studies, No. (%)
Type of treatment	
AT and PP vs. SAT and drugs	4 (16.67%)
Eacu vs. sham Eacu	6 (25%)
AT vs. SAT	15 (62.5%)
Acupoints location of CG	
Same as the IG	9 (37.5%)
Non-acupoints selected at X cm lateral to the Acupoint	13 (54.17%)
Non-acupoints selected at remote regions	2 (8.33%)
Needling instrument of CG	
Conventional disposable sterile acupuncture needles	14 (58.33%)
Blunt needle not inserted	8 (33.33%)
Retractable sham acupuncture	2 (8.33%)
Acupoints depth of CG	
Same as the IG	1 (4.17%)
Shallow puncture under the skin	11 (45.83%)
Not penetrated	12 (50%)
Manually manipulate of CG	
Yes	1 (4.17%)
None	23 (95.83%)
De qi of CG	
Yes	1 (4.17%)
None	18 (75.00%)
Not reported	5 (20.83%)

AT, Acupuncture Therapy; CG, Control group; Eacu, Electroacupuncture; IG, Intervention group; SAT, Sham Acupuncture Therapy; PP, Pharmacological Placebo.

### 3.2 Risk of bias within studies

Individual study risk of bias and summary risk of bias are presented in Table 1, Supplementary Table 3, and Supplementary Figure 1, respectively. Two studies (30, 44) were assessed as high risk primarily due to bias arising from missing outcome data, which was manifested explicitly in two aspects: a strong correlation between the missing outcome data and disease outcomes, as well as high dropout rates. Five (23, 29, 33, 38, 40) studies were evaluated as having some concerns regarding the mention of randomization only, without allocation concealment or blinding of outcome assessors. Moreover, twelve (18–20, 24–27, 29, 32, 34, 38–40, 42) studies lacked information on whether they followed the predefined analysis protocol, and two (35, 37) studies employed outcome measurement methods that diverged from previously established protocols; thus, we rated them as having some concerns about the risk of bias. Given the particularity of acupuncture operations, blinding of acupuncture therapists was impractical in these studies; consequently, it was deemed to have no impact on the study results. All studies were assessed as having a low risk of deviations from the intended interventions.

The detailed acupuncture treatment protocol for the included studies was evaluated by STRICTA (Supplementary Table 4). Nearly all studies reported acupuncture style (item 1a, 100%), reasoning for acupuncture treatment (item 1b, 100%), acupoints names (item 2b, 100%), retention time (item 2f, 95.83%), needle type (item 2g, 91.67%), treatment regimen (item 3a and 3b, 95.83%), and setting and context of treatment (item 4b, 100%). The three items with the most limited available data are the details of other interventions administered to the acupuncture group (item 4a, 25%), the extent to which treatment was varied (item 1c, 29.17%), and the background of acupuncturists (item 5, 41.67%).

### 3.3 Certainty of evidence and effect estimates

**Figure 2:**
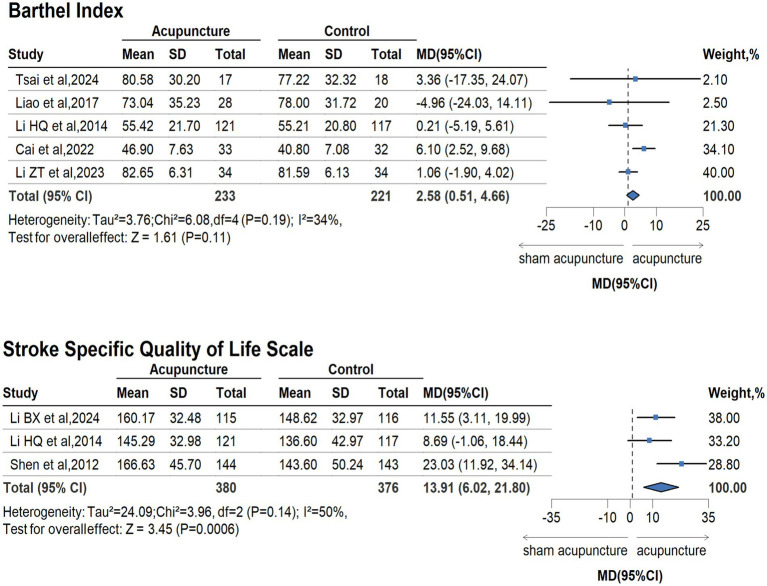
Forest plot for BI and SSQOL.

**Figure 3:**
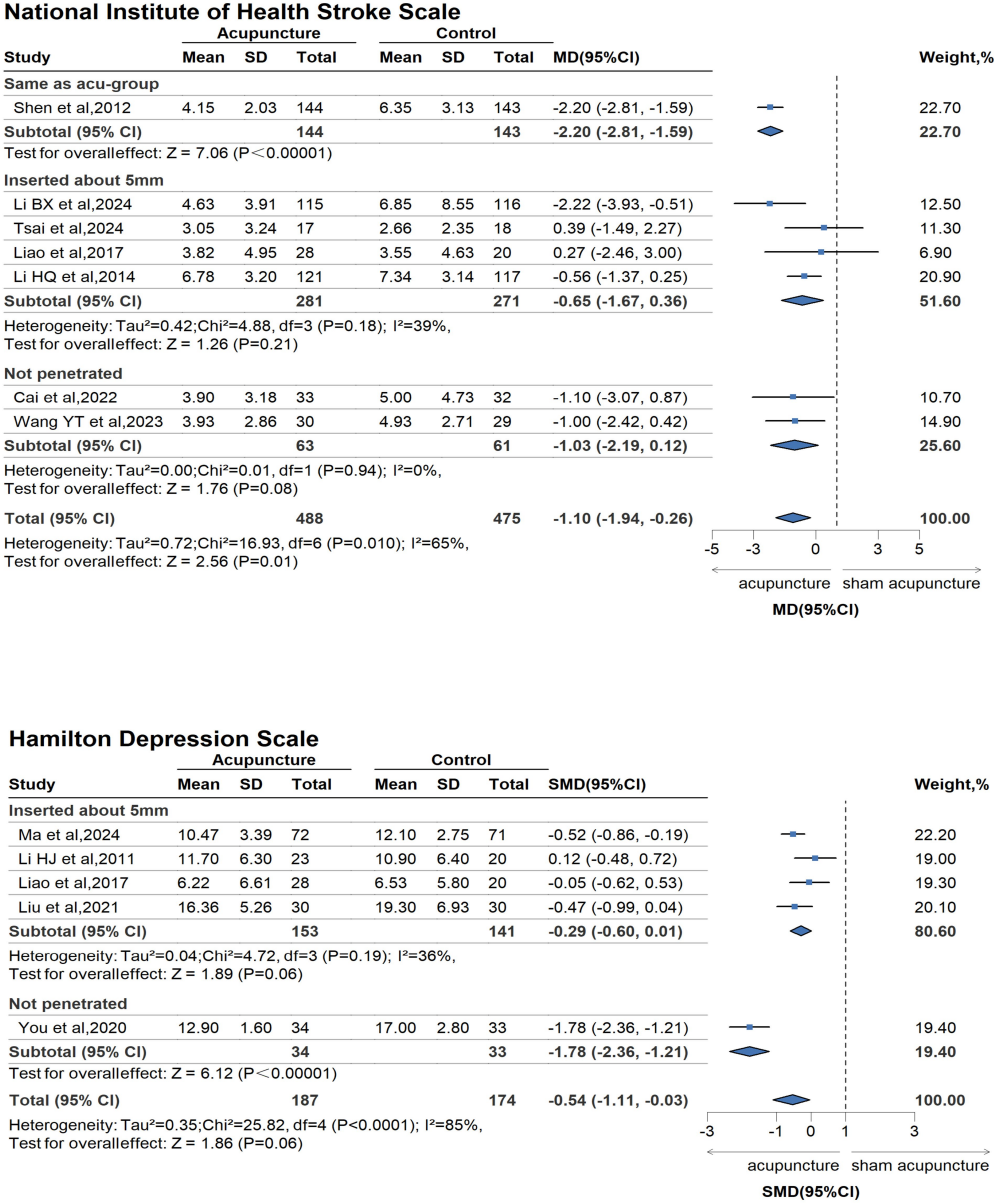
Forest plot of the subgroup analyses for NIHSS and HAMD.

The GRADE for available outcomes is presented in Supplementary Table 5. Compared with sham/placebo acupuncture for people with stroke and its complications, acupuncture had beneficial effects on the improvement of life quality (measured by SSQOL, 3 trails, 756 participants; MD 13.91, 95%CI 6.02 to 21.80; GRADE moderate), neurological defection (measured by NIHSS, 7 trails, 963 participants; MD −1.10, 95%CI −1.94 to −0.26; GRADE low), and depression [measured by Hamilton depression scale (HAMD), 5 trails, 361 participants; SMD −0.54, 95%CI −1.11 to −0.03; GRADE low]. According to the plot, compared with the sham acupuncture group, acupuncture was associated with better improvement in neurological defection, life quality, and depression for stroke. There was no difference between sham acupuncture and acupuncture in the daily living ability (measured by BI, 5 trails, 454 participants; MD 2.58, 95%CI 0.51 to 4.66; GRADE low) (Figures 2, 3, Supplementary Table 5).

In the subgroup analyses for the sham/placebo-controlled design (Figure 3), the type of acupoint depth explained this heterogeneity. We divided the included studies into 3 groups based on the acupoint depth of the control group: the same as the acu-group, inserted about 5 mm, and not penetrated. The pooled findings of the neurological deficit, which was measured by NIHSS, favored the shallow puncture under the skin, approximately 5 mm (4 studies, MD −0.65; 95%CI, −1.67 to 0.36; I2 = 39%), and not penetrating (2 studies, MD −1.03; 95%CI, −2.19 to 0.12; I2 = 0%), all contributing to reduced heterogeneity. Meanwhile, the heterogeneity in HAMD scale results decreased when acupoints were punctured at a shallow depth of approximately 5 mm beneath the skin (4 studies, MD −0.29; 95%CI, −0.60 to 0.01; I2 = 36%). Subgroup analysis was not performed due to inadequate data from one RCT.

According to the results of the comparison of the effects of different sham acupuncture characteristics, a significant difference was found between acupoint depth, type of treatment, manual depth, and de qi (Supplementary Figure 2). Specifically, the correlation between different acupoint depths and efficacy has been validated through four assessment dimensions: BI, SSQOL, NIHSS, and HAMD. Except for BI scale findings, which showed no statistically significant difference between shallow puncture under the skin and acupuncture (MD 0.80; 95%CI, −1.75 to 3.35), all other assessments demonstrated statistically significant differences. The associations between different acupuncture details (treatment type, manually manipulation, and de qi sensation) and efficacy were validated through three (BI, NIHSS, HAMD), two (SSQOL, NIHSS), and two (SSQOL, NIHSS) assessment dimensions, respectively, all showing statistically significant differences when compared with true acupuncture. Hence, the acupoint depth, type of treatment, manual manipulation, and de qi sensation remain essential considerations in the design of sham acupuncture controls.

### 3.4 Adverse events

The adverse events, explicitly evidenced in the 14 studies conclusions, reported were minor and did not require any medical intervention or evaluation (Supplementary Figure 3). The most frequently observed adverse events included pain, bleeding, hematomas, and fainting after treatment. The reported adverse events showed either fewer occurrences or no significant differences between groups [Odds Ratio (OR) 1.20; 95%CI, 0.82 to 1.77, *p* = 0.36], as observed in comparisons of acupuncture combined with pharmacological placebo vs. sham acupuncture with drugs (8.42% vs. 8.06%), electroacupuncture vs. sham electroacupuncture (5.56% vs. 7.04%), and acupuncture vs. sham acupuncture (7.61% vs. 5.77%).

## 4 Discussion

This study aimed to evaluate the association between acupuncture therapy and stroke, as well as its complications. The selected studies included 24 RCTs involving 2,310 stroke patients. Although a vast number of original studies have investigated acupuncture for stroke and its complications, only a limited number employed sham /placebo-controlled designs. The results revealed an association between acupuncture and greater improvement in life quality, neurological defection, and depression in stroke patients, with a moderate to low level of certainty. Existing evidence primarily focuses on acupuncture as a complementary and alternative therapy, highlighting its benefits in managing stroke-related symptoms (9, 13, 41, 46), which are in line with the findings of this study. Most countries have already incorporated acupuncture into the symptomatic management of stroke patients. However, acupuncture may not be suitable as a standalone treatment for stroke and its complications (10, 47). Key factors such as the dose–response relationship of acupuncture and the half-life of its therapeutic effects remain insufficiently elucidated (48). Consequently, current evidence does not meet the precision medicine requirements for stroke patients, such as stable blood pressure control and antiplatelet therapy.

The quantification of element manipulation in acupuncture therapy varies, ranging from the site of intervention (acupoint selection, acupoints depth), timing factors (timing of intervention, retention duration, treatment course, and frequency), and techniques (manipulation methods, de qi sensation), all of which are critical to therapeutic efficacy (18–20, 49). Acupuncturists need information including specific operational methods, dosage, dose–response relationships, and overall effectiveness. This study identified significant variability in acupuncture treatment across different studies, particularly in the selection of acupoints and manipulation techniques. We postulated that these discrepancies may be attributed to the vast and intricate theory of Traditional Chinese Medicine (TCM), the diversity of acupuncture academic ideology (50–52), and the combinatorial variability of acupuncture manipulation quantification. Moreover, it is reasonable to adopt targeted treatment for different stroke complications. Consequently, significant heterogeneity was observed in included RCTs. Given that the actual effect size may vary across all studies, a random-effects model was employed in this study.

Additionally, inadequate reporting of acupuncture treatment was identified, which is in line with findings from previous systematic reviews (10, 53). However, none of the authors responded to inquiries regarding incomplete information. Insufficient reporting obstructed the summary of evidence (54) that may be attributable to a significant limiting factor in validating and spreading the efficacy of acupuncture. This underscores a necessity for more stringent requirements for acupuncture manipulation, researcher training, and the implementation of standardized reporting protocols.

This study compared the therapeutic effects of sham acupuncture (including placebo needles) with acupuncture. The results demonstrated that the acupuncture group showed significantly superior improvements in quality of life, neurological function, and depressive symptoms compared to the sham acupuncture group. The certainty of evidence for these estimates ranged from moderate to low. Sham acupuncture, a type of penetrating sham needle, differs from verum acupuncture primarily in terms of acupoint location, needling depth, and minimal stimulation intensity (55). Placebo needles are typically designed with blunt or telescopic needles to prevent skin penetration (56, 57). For sham electroacupuncture, minimal or no electrical current is applied as a control method (45, 58). Evidence from this study revealed that variations in needling depth, type, manual manipulation, and de qi sensation could lead to differential therapeutic effects. This finding suggests that the therapeutic significance observed after acupuncture therapy may be mainly due to these specific elements of acupuncture therapy. Therefore, further investigation into the physiological mechanisms underlying acupuncture and sham acupuncture is needed to enhance the credibility of acupuncture.

Blinding is a critical factor influencing the reliability of evidence and the applicability of conclusions (59). This study mitigated the limitations of low-quality and highly heterogeneous meta-analysis results by including studies with single- or double-blind designs. However, due to the complex physiological nature of acupuncture, achieving fully effective blinding or designing an appropriate sham control remains challenging (55), which still impacts the evaluation of evidence quality. For sham acupuncture, needling at the same acupoints cannot be considered a true placebo control (60), as the acupoint position itself exerts physiological effects that correlate with clinical outcomes. For placebo needles, when both the acupuncture and sham groups utilize the same basic equipment, the risk of unblinding is relatively low (60). Clinical studies employing a double-blind, double-simulated design (acupuncture plus placebo versus sham acupuncture plus drugs) demonstrate better blinding assessment status. Among the included studies, the double-blind, double-simulated design was primarily applied in clinical research on PSD. This remains the only design capable of blinding acupuncturists. These findings suggest that continuous monitoring of blinding status and transparent reporting of blinding assessments could further optimize sham controls and effectively mitigate blinding bias.

Adverse effects associated with acupuncture were infrequent, with no significant difference observed between the acupuncture and sham groups, consistent with previous findings (10). Acupuncture therapy is particularly valuable for patients with recurrent or refractory conditions. Stroke, as one such condition, is characterized by high disability, recurrence, and mortality rates (61), necessitating close attention to long-term management and further exploration of the relationship between acupuncture treatment dosage and its efficacy and safety.

This study had several limitations. First, the number of included studies was limited. Only studies published after 2000 from 7 Chinese and English databases were incorporated, which may have resulted in omissions. Second, due to the diversity of acupuncture manipulation, it is not possible to provide a conclusive optimal acupuncture strategy or optimal sham needle design. Third, as the included studies contained various stroke complications, many clinical outcomes were reported in only a small number. This not only restricted the evaluation of acupuncture effect estimates but also limited the analysis of efficacy differences among various sham acupuncture design elements. Fourth, the assessment of placebo effects was constrained by the absence of waitlist control groups. Fifth, the general lack of blinding of acupuncturists and outcome assessors, which is a common challenge in acupuncture RCTs.

## 5 Conclusion

The findings of this study demonstrate that acupuncture is associated with improved life quality, neurological function, and depressive symptoms in stroke patients (with moderate- or low-quality certainty evidence), and its efficacy is significantly better than sham acupuncture. In this systematic review and meta-analysis, variations in needling depth, type, manual manipulation, and de qi sensation were examined in relation to outcomes. These findings suggest that future acupuncture research should prioritize these key elements and explore optimal acupuncture strategies and sham needle design methodologies to enhance the effectiveness of acupuncture.

## Data Availability

The original contributions presented in the study are included in the article/Supplementary material, further inquiries can be directed to the corresponding author.

## References

[1] Collaborators, GBD 2019 Stroke. Global, regional, and national burden of stroke and its risk factors, 1990–2019: a systematic analysis for the global burden of disease study 2019. Lancet Neurol. (2021) 20:795–820. doi: 10.1016/S1474-4422(21)00252-0, PMID: 34487721 PMC8443449

[2] Scott CA Li LX Rothwell PM. Diverging temporal trends in stroke incidence in younger vs older people: a systematic review and meta-analysis. JAMA Neurol. (2022) 79:1036–48. doi: 10.1001/jamaneurol.2022.1520, PMID: 35943738 PMC9364236

[3] Martin SS Aday AW Allen NB Almarzooq ZI Anderson CAM Arora P . 2025 heart disease and stroke statistics: a report of US and global data from the American Heart Association. Circulation. (2025) 151:e41–e660. doi: 10.1161/cir.0000000000001303 39866113 PMC12256702

[4] Wafa HA Wolfe CDA Emmett E Roth GA Johnson CO Wang YZ. Burden of stroke in Europe. Stroke. (2020) 51:2418–27. doi: 10.1161/strokeaha.120.029606, PMID: 32646325 PMC7382540

[5] Gimigliano F Negrini S. The World Health Organization "rehabilitation 2030: a call for action". Eur J Phys Rehabil Med. (2017) 53:155–68. doi: 10.23736/S1973-9087.17.04746-3, PMID: 28382807

[6] Wu P Mills E Moher D Seely D. Acupuncture in Poststroke rehabilitation: a systematic review and Meta-analysis of randomized trials. Stroke. (2010) 41:e171–9. doi: 10.1161/STROKEAHA.109.573576 20167912

[7] Cao BQ Tan F Zhan J Lai PH. Mechanism underlying treatment of ischemic stroke using acupuncture: transmission and regulation. Neural Regen Res. (2021) 16:944–54. doi: 10.4103/1673-5374.297061, PMID: 33229734 PMC8178780

[8] Jiang HL Zhang C Lin MX Yin Y Deng SZ Liu W . Deciphering the mechanistic impact of acupuncture on the neurovascular unit in acute ischemic stroke: insights from basic research in a narrative review. Ageing Res Rev. (2024) 101:102536. doi: 10.1016/j.arr.2024.102536 39384155

[9] Zhang S Wu B Liu M Li N Zeng X Liu H . Acupuncture efficacy on ischemic stroke recovery: multicenter randomized controlled trial in China. Stroke. (2015) 46:1301–6. doi: 10.1161/STROKEAHA.114.007659, PMID: 25873601

[10] Yang A Wu HM Tang JL Xu L Yang M Liu GJ. Acupuncture for stroke rehabilitation. Cochrane Database Syst Rev. (2016) 2016:CD004131. doi: 10.1002/14651858.CD004131.pub3, PMID: 27562656 PMC6464684

[11] Chae Y Chang DS Lee SH Jung WM Lee IS Jackson S . Inserting needles into the body: a meta-analysis of brain activity associated with acupuncture needle stimulation. J Pain. (2013) 14:215–22. doi: 10.1016/j.jpain.2012.11.011, PMID: 23395475

[12] Zhang YQ Zhang HL Nierhaus T Pach D Witt CM Yi M. Default mode network as a neural substrate of acupuncture: evidence, challenges and strategy. Front Neurosci. (2019) 13:100. doi: 10.3389/fnins.2019.00100, PMID: 30804749 PMC6378290

[13] NIH. NIH consensus conference. Acupuncture Jama. (1998) 280:1518–24. doi: 10.1001/jama.280.17.1518 9809733

[14] WHO. WHO global report on traditional and complementary medicine 2019. Geneva: World Health Organization (2019).

[15] Xu M Li D Zhang S. Acupuncture for acute stroke. Cochrane Database Syst Rev. (2018) 3:CD003317. doi: 10.1002/14651858.CD003317.pub3, PMID: 29607495 PMC6956658

[16] Birch S Robinson N. Acupuncture as a post-stroke treatment option: a narrative review of clinical guideline recommendations. Phytomedicine. (2022) 104:154297. doi: 10.1016/j.phymed.2022.154297, PMID: 35816994

[17] Stinear CM Lang CE Zeiler S Byblow WD. Advances and challenges in stroke rehabilitation. Lancet Neurol. (2020) 19:348–60. doi: 10.1016/S1474-4422(19)30415-6, PMID: 32004440

[18] Wang XY Liu W Li H Rong MY Li JY Wang SK . Effectiveness of acupuncture treatment for stroke and stroke complications: a protocol for meta-analysis and systematic review based on randomized, single-blind, controlled trials. Front Neurol. (2023a) 14:1255999. doi: 10.3389/fneur.2023.1255999, PMID: 38020598 PMC10651727

[19] Wang YT Li MC Li KY Xu XY Zhuang LX. Standardized Jin's three-needle therapy for stroke: a randomized controlled trial. Zhongguo Zhen Jiu. (2023b) 43:9–13. doi: 10.13703/j.0255-2930.20220415-k0003, PMID: 36633232

[20] Wang BG Xu LL Yang HY Xie J Xu G Tang WC. Manual acupuncture for neuromusculoskeletal disorders: the selection of stimulation parameters and corresponding effects. Front Neurosci. (2023c) 17:1096339. doi: 10.3389/fnins.2023.1096339 36793537 PMC9922711

[21] GRADE (2025). Grading of Recommendations Assessment, Development, and Evaluations (GRADE) Working Group. Available online at: https://www.Gradeworkinggroup.org/ (Accessed March 10, 2025).

[22] Yoshida M Kinoshita Y Watanabe M Sugano K. JSGE clinical practice guidelines 2014: standards, methods, and process of developing the guidelines. J Gastroenterol. (2015) 50:4–10. doi: 10.1007/s00535-014-1016-1, PMID: 25448314

[23] Lee SY Baek YH Park SU Moon SK Park JM Kim YS . Intradermal acupuncture on shen-men and nei-kuan acupoints improves insomnia in stroke patients by reducing the sympathetic nervous activity – a randomized clinical trial. Am J Chin Med. (2009) 37:1013–21. doi: 10.1142/S0192415X09007624, PMID: 19938212

[24] Li HJ Zhong BL Fan YP Hu HT. Acupuncture for post-stroke depression: a randomized controlled trail. Zhongguo Zhen Jiu. (2011) 31:3–6. doi: 10.13703/j.0255-2930.2011.01.002 21355143

[25] Zheng QH Yu B Li Y Yang ZQ Yu SY. Clinical study of acupuncture with language training for aphasia after stroke. J Liaoning Univ TCM. (2011) 13:105–7. doi: 10.13194/j.jlunivtcm.2011.01.107.zhengqh.086

[26] Shen PF Kong L Ni LW Guo HL Yang S Zhang LL . Acupuncture intervention in ischemic stroke: a randomized controlled prospective study. Am J Chin Med. (2012) 40:685–93. doi: 10.1142/s0192415x12500516, PMID: 22809024

[27] Liu Y Liu LR Wang XM. Electroacupuncture at points Baliao and Huiyang (BL35) for post-stroke detrusor overactivity. Neural Regen Res. (2013) 8:1663–72. doi: 10.3969/j.issn.1673-5374.2013.18.004, PMID: 25206463 PMC4145909

[28] Li HQ Liu HL Liu CZ Shi GX Zhou W Zhao CM . Effect of “Deqi” during the study of needling “Wang’s Jiaji” acupoints treating spasticity after stroke. Evid Based Complement Alternat Med. (2014) 2014:715351. doi: 10.1155/2014/715351, PMID: 25477996 PMC4247953

[29] Qian XL Zhou X You YL Shu S Fang FF Huang SR . Traditional Chinese acupuncture for poststroke depression: a single-blind double-simulated randomized controlled trial. J Altern Complement Med. (2015) 21:748–53. doi: 10.1089/acm.2015.0084, PMID: 26383034

[30] Xia WG Zheng CJ Xia JH Zhang YP. Post-stroke dysphagia treated with acupuncture of meridian differentiation: a randomized controlled trail. Zhongguo Zhen Jiu. (2016) 36:673–8. doi: 10.13703/j.0255-2930.2016.07.001 29231403

[31] Liao HY Ho WC Chen CC Lin JG Chang CC Chen LY . Clinical evaluation of acupuncture as treatment for complications of cerebrovascular accidents: a randomized, sham-controlled, subject- and Assessor-blind trial. Evid Based Complement Alternat Med. (2017) 2017:7498763. doi: 10.1155/2017/7498763, PMID: 28408941 PMC5376930

[32] Xiong J Zhang ZC Ma Y Li ZH Zhou F Qiao N . The effect of combined scalp acupuncture and cognitive training in patients with stroke on cognitive and motor functions. NeuroRehabilitation. (2020) 46:75–82. doi: 10.3233/nre-192942, PMID: 32039871

[33] You YL Zhang TF Shu S Qian XL Zhou S Yao F. Wrist-ankle acupuncture and fluoxetine in the treatment of post-stroke depression a randomized controlled clinical trial. J Tradit Chin Med. (2020) 40:455–60. doi: 10.19852/j.cnki.jtcm.2020.03.014 32506860

[34] Liu Q Chen J Wu T Tian Q Wang Y. Clinical study of Tiaoxin Anshen acupuncture in treating post-stroke depression. J Clin Acupunct moxibustion. (2021) 37:24–8. doi: 10.19917/j.cnki.1005-0779.021134

[35] Cai W Ma W Li YJ Wang GT Yang H Shen WD. Efficacy and safety of electroacupuncture for post-stroke depression: a randomized controlled trial. Acupunct Med. (2022) 40:434–42. doi: 10.1177/09645284221077104, PMID: 35232229

[36] Cao Y Yan YJ Xu JY Liwayiding A Liu YP Yin X . Acupuncture for insomnia after ischemic stroke: an assessor-participant blinded, randomized controlled trial. Acupunct Med. (2022) 40:443–52. doi: 10.1177/09645284221077106, PMID: 35317665

[37] Li LC Wang XJ Guo JX Chen YL Wang ZY. Effect of acupuncture in the acute phase of intracerebral hemorrhage on the prognosis and serum BDNF: a randomized controlled trial. Front Neurosci. (2023a) 17:1167620. doi: 10.3389/fnins.2023.1167620, PMID: 37123377 PMC10133506

[38] Li T Jin XQ Zhang BX Dong Y Deng L Lin LJ . The clinical efficacy of acupuncture treatment after cerebral infarction dysphagia based on ultrasound measurement of hyoid bone - thyroid cartilage movement distance. Guangdong Med J. (2023b) 44:766–72. doi: 10.13820/j.cnki.gdyx.20225411

[39] Li ZT Ban LQ Chen F. Acupuncture of revised acupoint combination around the skull base for post-stroke mild cognitive impairment: a randomized controlled trial. Zhongguo Zhen Jiu. (2023c) 43:1104–8. doi: 10.13703/j.0255-2930.20221231-0002, PMID: 37802513

[40] Zheng C Wu WB Fan DF Lian QQ Guo F Tang LL. Acupuncture' effect on nerve remodeling among patients with dysphagia after cerebral infraction: a study based on diffusion tensor imaging. World J Acupunct-Moxibustion. (2023) 33:118–25. doi: 10.1016/j.wjam.2022.12.003

[41] Li BX Deng SZ Zhuo BF Sang BM Chen JJ Zhang ML . Effect of acupuncture vs sham acupuncture on patients with Poststroke motor aphasia. JAMA Netw Open. (2024a) 7, 1–13. doi: 10.1001/jamanetworkopen.2023.52580 PMC10804271 38252438

[42] Li MC Wang YT Li KY Xu XY Zhuang LX. Efficacy of the spirit-regulation method of Jin’s three-needle therapy for post-stroke anxiety and its effect on the hypothalamus-pituitary-adrenal axis. Zhen Ci Yan Jiu. (2024b) 49:57–63. doi: 10.13702/j.1000⁃0607.20220949 38239139

[43] Ma FX Cao GP Lu L Zhu YL Li WL Chen L. Electroacupuncture versus escitalopram for mild to moderate post-stroke depression: a randomized non-inferiority trial. Front Psych. (2024) 15:1332107. doi: 10.3389/fpsyt.2024.1332107, PMID: 38370556 PMC10869574

[44] Tsai CY Liao WL Wu HM Chang CW Chen WL Hsieh CL. Acupuncture improves neurological function and anti-inflammatory effect in patients with acute ischemic stroke: a double-blinded randomized controlled trial. Complement Ther Med. (2024) 82:103049. doi: 10.1016/j.ctim.2024.103049, PMID: 38729273

[45] Zhang BY Zhou Y Feng LY Sui D He L Tong D . A neural regulation mechanism of head electroacupuncture on brain network of patients with stroke related sleep disorders. J Tradit Chin Med. (2024) 44:1268–76. doi: 10.19852/j.cnki.jtcm.2024.06.011 39617712 PMC11589561

[46] Wang LP Su XT Yang NN Wang QY Yang JW Liu CZ. Electroacupuncture improves cerebral blood flow in pMCAO rats during acute phase via promoting leptomeningeal collaterals. J Cereb Blood Flow Metab. (2025) 45:1507–18. doi: 10.1177/0271678X241270240, PMID: 40007441 PMC11863195

[47] Liu XL Qian ZD Li YX Wang YW Zhang Y Zhang Y . Unveiling synergies: integrating TCM herbal medicine and acupuncture with conventional approaches in stroke management. Neuroscience. (2025) 567:109–22. doi: 10.1016/j.neuroscience.2024.12.043, PMID: 39730019

[48] Yoon DE Lee IS Chae Y. Identifying dose components of manual acupuncture to determine the dose-response relationship of acupuncture treatment: a systematic review. Am J Chin Med. (2022) 50:653–71. doi: 10.1142/S0192415X22500264, PMID: 35300569

[49] Wang MY Zhang SJ Zhang XB Tian YR Sun YX. Standardization studies of acupuncture prescriptions: a new approach to translational medicine in acupuncture. Zhongguo Zhen Jiu. (2024) 44:94–8. doi: 10.13703/j.0255-2930.20230505-0003 38191166

[50] Li HQ Long DH Li BX Liu HL Ma TT Wu TY . A clinical study to assess the influence of acupuncture at “Wang’s Jiaji” acupoints on limb spasticity in patients in convalescent stage of ischemic stroke: study protocol for a randomized controlled trial. Trials. (2019) 20:419. doi: 10.1186/s13063-019-3464-7, PMID: 31291976 PMC6621988

[51] Yang XJ Yu HB Zhang T Luo X Ding L Chen B . The effects of Jin's three-needle acupuncture therapy on EEG alpha rhythm of stroke patients. Top Stroke Rehabil. (2018) 25:535–539. doi: 10.1080/10749357.2018.1484680 30599806

[52] Song ZT Huang QY Guo YX Song XD Zhang XB Xiao HJ. Xingnao Kaiqiao acupuncture method combined with temporal three-needle in the treatment of acute ischemic stroke: a randomized controlled trial. Comput Intell Neurosci. (2022) 2022:8145374. doi: 10.1155/2022/8145374 35814561 PMC9259275

[53] Cai YY Zhang SQ Liu SN Wen ZH Xue CC. Electroacupuncture for poststroke spasticity: a systematic review and meta-analysis. Arch Phys Med Rehabil. (2017) 98:2578–89. doi: 10.1016/j.apmr.2017.03.023 28455191

[54] Svenkerud S Macpherson H. The impact of STRICTA and CONSORT on reporting of randomised control trials of acupuncture: a systematic methodological evaluation. Acupunct Med. (2018) 36:349–57. doi: 10.1136/acupmed-2017-011519, PMID: 30201785

[55] Liu TL Jiang LJ Li SJ Cheng SY Zhuang R Xiong ZY . The blinding status and characteristics in acupuncture clinical trials: a systematic reviews and meta-analysis. Syst Rev. (2024) 13:302. doi: 10.1186/s13643-024-02692-0, PMID: 39643890 PMC11624600

[56] Streitberger AK Kleinhenz ADJ. Introducing a placebo needle into acupuncture research. Lancet. (1998) 352:364–5. doi: 10.1016/S0140-6736(97)10471-8, PMID: 9717924

[57] Enblom A Johnsson A Hammar M Onelöv E Steineck G Börjeson S. Acupuncture compared with placebo acupuncture in radiotherapy-induced nausea-a randomized controlled study. Ann Oncol. (2012) 23:1353–61. doi: 10.1093/annonc/mdr402, PMID: 21948812

[58] Kong JT Puetz C Tian L Haynes I Mackey S. Effect of electroacupuncture vs sham treatment on change in pain severity among adults with chronic low back pain: a randomized clinical trial. JAMA Netw Open. (2020) 3:e2022787. doi: 10.1001/jamanetworkopen.2020.22787, PMID: 33107921 PMC7592030

[59] Boutron I Estellat C Guittet L Dechartres A Sackett DL Hróbjartsson A . Methods of blinding in reports of randomized controlled trials assessing pharmacologic treatments: a systematic review. PLoS Med. (2006) 3:e425. doi: 10.1371/journal.pmed.0030425, PMID: 17076559 PMC1626553

[60] Lee B Kwon CY Lee HW Nielsen A Wieland LS Kim TH . Needling point location used in sham acupuncture for chronic nonspecific low back pain: a systematic review and network meta-analysis. JAMA Netw Open. (2023) 6:e2332452. doi: 10.1001/jamanetworkopen.2023.32452, PMID: 37672270 PMC10483312

[61] Hilkens NA Casolla B Leung TW DeLeeuw FE. Stroke Lancet. (2024) 403:2820–36. doi: 10.1016/S0140-6736(24)00642-1 38759664

