# Supplementary material for: Use of acupuncture in stroke and stroke complications: a systematic review and meta-analysis based on sham-controlled trials
Source: Front Neurol. 2025 Oct 22;16:1668497. doi: 10.3389/fneur.2025.1668497 (PMC12586079; doi:10.3389/fneur.2025.1668497)

Supplementary Material

Contents

[1 Supplementary Appendix 1. Search strategy 2](#_Toc203726029)

[2 Supplementary Table 1. Characteristics of patients and treatment time details included in studies. 9](#_Toc203726030)

[3 Supplementary Table 2. Details of acupuncture treatment in intervention group vs control group. 11](#_Toc203726031)

[4 Supplementary Table 3. Study risk of bias. 15](#_Toc203726032)

[5 Supplementary Table 4. STRICTA checklist for the included studies. 18](#_Toc203726033)

[6 Supplementary Table 5. Summary of findings for the main comparison. 20](#_Toc203726034)

[7 Supplementary Figure 1. Risk of bias graph: each risk of bias item presented as percentages across all included studies. 21](#_Toc203726035)

[8 Supplementary Figure 2. Comparison of the effects of different sham acupuncture. 22](#_Toc203726036)

[9 Supplementary Figure 3. Summary of the adverse. 23](#_Toc203726037)

Supplementary Appendix 1. Search strategy

**PubMed:**

| #6 | Search: **((#1) OR (#2) OR (#3)) AND (#4) AND (#5)** Sort by: **Most Recent** | [92](https://pubmed.ncbi.nlm.nih.gov/?term=%28%28%231%29+OR+%28%232%29+OR+%28%233%29%29+AND+%28%234%29+AND+%28%235%29&sort=&size=200) | 21:05:54 |
| --- | --- | --- | --- |
| #5 | Search: **(((((((((((((sham acupuncture[Title/Abstract]) OR (placebo acupuncture[Title/Abstract])) OR (non-acupoint acupuncture[Title/Abstract])) OR (Nonacupoints[Title/Abstract])) OR (non-meridian points[Title/Abstract])) OR (shallow acupuncture[Title/Abstract])) OR (minimal acupuncture[Title/Abstract])) OR (Superficial acupuncture[Title/Abstract])) OR (acupoints acupuncture therapy[Title/Abstract])) OR (low-sensitized points[Title/Abstract])) OR (non-sensitized points[Title/Abstract])) OR (placebo acupoint[Title/Abstract])) OR (basic acupuncture[Title/Abstract])) OR (non-affected meridian acupuncture[Title/Abstract])** Sort by: **Most Recent** | [2,920](https://pubmed.ncbi.nlm.nih.gov/?term=%28%28%28%28%28%28%28%28%28%28%28%28%28sham+acupuncture%5BTitle%2FAbstract%5D%29+OR+%28placebo+acupuncture%5BTitle%2FAbstract%5D%29%29+OR+%28non-acupoint+acupuncture%5BTitle%2FAbstract%5D%29%29+OR+%28Nonacupoints%5BTitle%2FAbstract%5D%29%29+OR+%28non-meridian+points%5BTitle%2FAbstract%5D%29%29+OR+%28shallow+acupuncture%5BTitle%2FAbstract%5D%29%29+OR+%28minimal+acupuncture%5BTitle%2FAbstract%5D%29%29+OR+%28Superficial+acupuncture%5BTitle%2FAbstract%5D%29%29+OR+%28acupoints+acupuncture+therapy%5BTitle%2FAbstract%5D%29%29+OR+%28low-sensitized+points%5BTitle%2FAbstract%5D%29%29+OR+%28non-sensitized+points%5BTitle%2FAbstract%5D%29%29+OR+%28placebo+acupoint%5BTitle%2FAbstract%5D%29%29+OR+%28basic+acupuncture%5BTitle%2FAbstract%5D%29%29+OR+%28non-affected+meridian+acupuncture%5BTitle%2FAbstract%5D%29&sort=&size=200) | 21:04:31 |
| #4 | Search: **(((acupuncture[MeSH Major Topic]) OR (acupunctur*[MeSH Major Topic])) OR (acupoint*[MeSH Major Topic])) OR (needl*[MeSH Major Topic])** Sort by: **Most Recent** | [40,896](https://pubmed.ncbi.nlm.nih.gov/?term=%28%28%28acupuncture%5BMeSH+Major+Topic%5D%29+OR+%28acupunctur%2A%5BMeSH+Major+Topic%5D%29%29+OR+%28acupoint%2A%5BMeSH+Major+Topic%5D%29%29+OR+%28needl%2A%5BMeSH+Major+Topic%5D%29&sort=&size=200) | 21:04:13 |
| #3 | Search: **(((((((((((((((((((((((Cerebral Hemorrhage[MeSH Major Topic]) OR (Hemorrhage, Cerebrum[MeSH Major Topic])) OR (Cerebrum Hemorrhage[MeSH Major Topic])) OR (Cerebrum Hemorrhages[MeSH Major Topic])) OR (Hemorrhages, Cerebrum[MeSH Major Topic])) OR (Cerebral Parenchymal Hemorrhage[MeSH Major Topic])) OR (Cerebral Parenchymal Hemorrhages[MeSH Major Topic])) OR (Hemorrhage, Cerebral Parenchymal[MeSH Major Topic])) OR (Hemorrhages, Cerebral Parenchymal[MeSH Major Topic])) OR (Parenchymal Hemorrhage, Cerebral[MeSH Major Topic])) OR (Parenchymal Hemorrhages, Cerebral[MeSH Major Topic])) OR (Intracerebral Hemorrhage[MeSH Major Topic])) OR (Hemorrhage, Intracerebral[MeSH Major Topic])) OR (Hemorrhages, Intracerebral[MeSH Major Topic])) OR (Intracerebral Hemorrhages[MeSH Major Topic])) OR (Hemorrhage, Cerebral[MeSH Major Topic])) OR (Cerebral Hemorrhages[MeSH Major Topic])) OR (Hemorrhages, Cerebral[MeSH Major Topic])) OR (Brain Hemorrhage, Cerebral[MeSH Major Topic])) OR (Brain Hemorrhages, Cerebral[MeSH Major Topic])) OR (Cerebral Brain Hemorrhage[MeSH Major Topic])) OR (Cerebral Brain Hemorrhages[MeSH Major Topic])) OR (Hemorrhage, Cerebral Brain[MeSH Major Topic])) OR (Hemorrhages, Cerebral Brain[MeSH Major Topic])** Sort by: **Most Recent** | [25,127](https://pubmed.ncbi.nlm.nih.gov/?term=%28%28%28%28%28%28%28%28%28%28%28%28%28%28%28%28%28%28%28%28%28%28%28Cerebral+Hemorrhage%5BMeSH+Major+Topic%5D%29+OR+%28Hemorrhage%2C+Cerebrum%5BMeSH+Major+Topic%5D%29%29+OR+%28Cerebrum+Hemorrhage%5BMeSH+Major+Topic%5D%29%29+OR+%28Cerebrum+Hemorrhages%5BMeSH+Major+Topic%5D%29%29+OR+%28Hemorrhages%2C+Cerebrum%5BMeSH+Major+Topic%5D%29%29+OR+%28Cerebral+Parenchymal+Hemorrhage%5BMeSH+Major+Topic%5D%29%29+OR+%28Cerebral+Parenchymal+Hemorrhages%5BMeSH+Major+Topic%5D%29%29+OR+%28Hemorrhage%2C+Cerebral+Parenchymal%5BMeSH+Major+Topic%5D%29%29+OR+%28Hemorrhages%2C+Cerebral+Parenchymal%5BMeSH+Major+Topic%5D%29%29+OR+%28Parenchymal+Hemorrhage%2C+Cerebral%5BMeSH+Major+Topic%5D%29%29+OR+%28Parenchymal+Hemorrhages%2C+Cerebral%5BMeSH+Major+Topic%5D%29%29+OR+%28Intracerebral+Hemorrhage%5BMeSH+Major+Topic%5D%29%29+OR+%28Hemorrhage%2C+Intracerebral%5BMeSH+Major+Topic%5D%29%29+OR+%28Hemorrhages%2C+Intracerebral%5BMeSH+Major+Topic%5D%29%29+OR+%28Intracerebral+Hemorrhages%5BMeSH+Major+Topic%5D%29%29+OR+%28Hemorrhage%2C+Cerebral%5BMeSH+Major+Topic%5D%29%29+OR+%28Cerebral+Hemorrhages%5BMeSH+Major+Topic%5D%29%29+OR+%28Hemorrhages%2C+Cerebral%5BMeSH+Major+Topic%5D%29%29+OR+%28Brain+Hemorrhage%2C+Cerebral%5BMeSH+Major+Topic%5D%29%29+OR+%28Brain+Hemorrhages%2C+Cerebral%5BMeSH+Major+Topic%5D%29%29+OR+%28Cerebral+Brain+Hemorrhage%5BMeSH+Major+Topic%5D%29%29+OR+%28Cerebral+Brain+Hemorrhages%5BMeSH+Major+Topic%5D%29%29+OR+%28Hemorrhage%2C+Cerebral+Brain%5BMeSH+Major+Topic%5D%29%29+OR+%28Hemorrhages%2C+Cerebral+Brain%5BMeSH+Major+Topic%5D%29&sort=&size=200) | 21:03:56 |
| #2 | Search: **((((((((((((((((((((((((Cerebral Infarctions[MeSH Major Topic]) OR (Infarctions, Cerebral[MeSH Major Topic])) OR (Infarction, Cerebral[MeSH Major Topic])) OR (Cerebral Infarct[MeSH Major Topic])) OR (Cerebral Infarcts[MeSH Major Topic])) OR (Infarct, Cerebral[MeSH Major Topic])) OR (Infarcts, Cerebral[MeSH Major Topic])) OR (Cerebral Infarction, Left Hemisphere[MeSH Major Topic])) OR (Left Hemisphere, Infarction, Cerebral[MeSH Major Topic])) OR (Infarction, Left Hemisphere, Cerebral[MeSH Major Topic])) OR (Left Hemisphere, Cerebral Infarction[MeSH Major Topic])) OR (Cerebral, Left Hemisphere, Infarction[MeSH Major Topic])) OR (Infarction, Cerebral, Left Hemisphere[MeSH Major Topic])) OR (Subcortical Infarction[MeSH Major Topic])) OR (Infarction, Subcortical[MeSH Major Topic])) OR (Infarctions, Subcortical[MeSH Major Topic])) OR (Subcortical Infarctions[MeSH Major Topic])) OR (Posterior Choroidal Artery Infarction[MeSH Major Topic])) OR (Anterior Choroidal Artery Infarction[MeSH Major Topic])) OR (Cerebral Infarction, Right Hemisphere[MeSH Major Topic])) OR (Right Hemisphere, Cerebral Infarction[MeSH Major Topic])) OR (Infarction, Right Hemisphere, Cerebral[MeSH Major Topic])) OR (Right Hemisphere, Infarction, Cerebral[MeSH Major Topic])) OR (Cerebral, Right Hemisphere, Infarction[MeSH Major Topic])) OR (Infarction, Cerebral, Right Hemisphere[MeSH Major Topic])** Sort by: **Most Recent** | [22,631](https://pubmed.ncbi.nlm.nih.gov/?term=%28%28%28%28%28%28%28%28%28%28%28%28%28%28%28%28%28%28%28%28%28%28%28%28Cerebral+Infarctions%5BMeSH+Major+Topic%5D%29+OR+%28Infarctions%2C+Cerebral%5BMeSH+Major+Topic%5D%29%29+OR+%28Infarction%2C+Cerebral%5BMeSH+Major+Topic%5D%29%29+OR+%28Cerebral+Infarct%5BMeSH+Major+Topic%5D%29%29+OR+%28Cerebral+Infarcts%5BMeSH+Major+Topic%5D%29%29+OR+%28Infarct%2C+Cerebral%5BMeSH+Major+Topic%5D%29%29+OR+%28Infarcts%2C+Cerebral%5BMeSH+Major+Topic%5D%29%29+OR+%28Cerebral+Infarction%2C+Left+Hemisphere%5BMeSH+Major+Topic%5D%29%29+OR+%28Left+Hemisphere%2C+Infarction%2C+Cerebral%5BMeSH+Major+Topic%5D%29%29+OR+%28Infarction%2C+Left+Hemisphere%2C+Cerebral%5BMeSH+Major+Topic%5D%29%29+OR+%28Left+Hemisphere%2C+Cerebral+Infarction%5BMeSH+Major+Topic%5D%29%29+OR+%28Cerebral%2C+Left+Hemisphere%2C+Infarction%5BMeSH+Major+Topic%5D%29%29+OR+%28Infarction%2C+Cerebral%2C+Left+Hemisphere%5BMeSH+Major+Topic%5D%29%29+OR+%28Subcortical+Infarction%5BMeSH+Major+Topic%5D%29%29+OR+%28Infarction%2C+Subcortical%5BMeSH+Major+Topic%5D%29%29+OR+%28Infarctions%2C+Subcortical%5BMeSH+Major+Topic%5D%29%29+OR+%28Subcortical+Infarctions%5BMeSH+Major+Topic%5D%29%29+OR+%28Posterior+Choroidal+Artery+Infarction%5BMeSH+Major+Topic%5D%29%29+OR+%28Anterior+Choroidal+Artery+Infarction%5BMeSH+Major+Topic%5D%29%29+OR+%28Cerebral+Infarction%2C+Right+Hemisphere%5BMeSH+Major+Topic%5D%29%29+OR+%28Right+Hemisphere%2C+Cerebral+Infarction%5BMeSH+Major+Topic%5D%29%29+OR+%28Infarction%2C+Right+Hemisphere%2C+Cerebral%5BMeSH+Major+Topic%5D%29%29+OR+%28Right+Hemisphere%2C+Infarction%2C+Cerebral%5BMeSH+Major+Topic%5D%29%29+OR+%28Cerebral%2C+Right+Hemisphere%2C+Infarction%5BMeSH+Major+Topic%5D%29%29+OR+%28Infarction%2C+Cerebral%2C+Right+Hemisphere%5BMeSH+Major+Topic%5D%29&sort=&size=200) | 21:03:33 |
| #1 | Search: **((((((((((((((((((((((((((((stroke[MeSH Major Topic]) OR (strokes[MeSH Major Topic])) OR (Cerebrovascular Accident[MeSH Major Topic])) OR (Cerebrovascular Accidents[MeSH Major Topic])) OR (CVA Cerebrovascular Accident[MeSH Major Topic])) OR (CVAs Cerebrovascular Accident[MeSH Major Topic])) OR (Cerebrovascular Apoplexy[MeSH Major Topic])) OR (Apoplexy, Cerebrovascular[MeSH Major Topic])) OR (Vascular Accident, Brain[MeSH Major Topic])) OR (Brain Vascular Accident[MeSH Major Topic])) OR (Brain Vascular Accidents[MeSH Major Topic])) OR (Vascular Accidents, Brai[MeSH Major Topic])) OR (Cerebrovascular Stroke[MeSH Major Topic])) OR (Cerebrovascular Strokes[MeSH Major Topic])) OR (Stroke, Cerebrovascular[MeSH Major Topic])) OR (Strokes, Cerebrovascular[MeSH Major Topic])) OR (Apoplexy[MeSH Major Topic])) OR (Cerebral Stroke[MeSH Major Topic])) OR (Cerebral Strokes[MeSH Major Topic])) OR (Stroke, Cerebral[MeSH Major Topic])) OR (Strokes, Cerebral[MeSH Major Topic])) OR (Stroke, Acute[MeSH Major Topic])) OR (Acute Stroke[MeSH Major Topic])) OR (Acute Strokes[MeSH Major Topic])) OR (Strokes, Acute[MeSH Major Topic])) OR (Cerebrovascular Accident, Acute[MeSH Major Topic])) OR (Acute Cerebrovascular Accident[MeSH Major Topic])) OR (Acute Cerebrovascular Accidents[MeSH Major Topic])) OR (Cerebrovascular Accidents, Acute[MeSH Major Topic])** Sort by: **Most Recent** | [148,002](https://pubmed.ncbi.nlm.nih.gov/?term=%28%28%28%28%28%28%28%28%28%28%28%28%28%28%28%28%28%28%28%28%28%28%28%28%28%28%28%28stroke%5BMeSH+Major+Topic%5D%29+OR+%28strokes%5BMeSH+Major+Topic%5D%29%29+OR+%28Cerebrovascular+Accident%5BMeSH+Major+Topic%5D%29%29+OR+%28Cerebrovascular+Accidents%5BMeSH+Major+Topic%5D%29%29+OR+%28CVA+Cerebrovascular+Accident%5BMeSH+Major+Topic%5D%29%29+OR+%28CVAs+Cerebrovascular+Accident%5BMeSH+Major+Topic%5D%29%29+OR+%28Cerebrovascular+Apoplexy%5BMeSH+Major+Topic%5D%29%29+OR+%28Apoplexy%2C+Cerebrovascular%5BMeSH+Major+Topic%5D%29%29+OR+%28Vascular+Accident%2C+Brain%5BMeSH+Major+Topic%5D%29%29+OR+%28Brain+Vascular+Accident%5BMeSH+Major+Topic%5D%29%29+OR+%28Brain+Vascular+Accidents%5BMeSH+Major+Topic%5D%29%29+OR+%28Vascular+Accidents%2C+Brai%5BMeSH+Major+Topic%5D%29%29+OR+%28Cerebrovascular+Stroke%5BMeSH+Major+Topic%5D%29%29+OR+%28Cerebrovascular+Strokes%5BMeSH+Major+Topic%5D%29%29+OR+%28Stroke%2C+Cerebrovascular%5BMeSH+Major+Topic%5D%29%29+OR+%28Strokes%2C+Cerebrovascular%5BMeSH+Major+Topic%5D%29%29+OR+%28Apoplexy%5BMeSH+Major+Topic%5D%29%29+OR+%28Cerebral+Stroke%5BMeSH+Major+Topic%5D%29%29+OR+%28Cerebral+Strokes%5BMeSH+Major+Topic%5D%29%29+OR+%28Stroke%2C+Cerebral%5BMeSH+Major+Topic%5D%29%29+OR+%28Strokes%2C+Cerebral%5BMeSH+Major+Topic%5D%29%29+OR+%28Stroke%2C+Acute%5BMeSH+Major+Topic%5D%29%29+OR+%28Acute+Stroke%5BMeSH+Major+Topic%5D%29%29+OR+%28Acute+Strokes%5BMeSH+Major+Topic%5D%29%29+OR+%28Strokes%2C+Acute%5BMeSH+Major+Topic%5D%29%29+OR+%28Cerebrovascular+Accident%2C+Acute%5BMeSH+Major+Topic%5D%29%29+OR+%28Acute+Cerebrovascular+Accident%5BMeSH+Major+Topic%5D%29%29+OR+%28Acute+Cerebrovascular+Accidents%5BMeSH+Major+Topic%5D%29%29+OR+%28Cerebrovascular+Accidents%2C+Acute%5BMeSH+Major+Topic%5D%29&sort=&size=200) | 21:02:50 |

**Embase:**

| #9 | #7 AND #8 | [122](https://www-embase-com-443.webvpn.cams.tsgvip.top/) |
| --- | --- | --- |
| #8 | #5 AND #6 | [3,003](https://www-embase-com-443.webvpn.cams.tsgvip.top/) |
| #7 | #1 OR #2 OR #3 OR #4 | [787,054](https://www-embase-com-443.webvpn.cams.tsgvip.top/) |
| #6 | 'sham acupuncture':ab,ti OR 'placebo acupuncture':ab,ti OR 'non-acupoint acupuncture':ab,ti OR 'nonacupoints':ab,ti OR 'non-meridian points':ab,ti OR 'shallow acupuncture':ab,ti OR 'minimal acupuncture':ab,ti OR 'superficial acupuncture':ab,ti OR 'acupoints acupuncture therapy':ab,ti OR 'low/non-sensitized points':ab,ti OR 'placebo acupoint':ab,ti OR 'basic acupuncture':ab,ti OR 'non-affected meridian acupuncture':ab,ti | [3,007](https://www-embase-com-443.webvpn.cams.tsgvip.top/) |
| #5 | 'acupuncture':ab,ti OR 'acupunctur*':ab,ti OR 'acupoint*':ab,ti OR 'needl*':ab,ti | [264,142](https://www-embase-com-443.webvpn.cams.tsgvip.top/) |
| #4 | 'hemorrhage, cerebrum':ab,ti OR 'cerebrum hemorrhage':ab,ti OR 'cerebrum hemorrhages':ab,ti OR 'hemorrhages, cerebrum':ab,ti OR 'cerebral parenchymal hemorrhage':ab,ti OR 'cerebral parenchymal hemorrhages':ab,ti OR 'hemorrhage, cerebral parenchymal':ab,ti OR 'hemorrhages, cerebral parenchymal':ab,ti OR 'parenchymal hemorrhage, cerebral':ab,ti OR 'parenchymal hemorrhages, cerebral':ab,ti OR 'intracerebral hemorrhage':ab,ti OR 'hemorrhage, intracerebral':ab,ti OR 'hemorrhages, intracerebral':ab,ti OR 'intracerebral hemorrhages':ab,ti OR 'hemorrhage, cerebral':ab,ti OR 'cerebral hemorrhages':ab,ti OR 'hemorrhages, cerebral':ab,ti OR 'brain hemorrhage, cerebral':ab,ti OR 'brain hemorrhages, cerebral':ab,ti OR 'cerebral brain hemorrhage':ab,ti OR 'cerebral brain hemorrhages':ab,ti OR 'hemorrhage, cerebral brain':ab,ti OR 'hemorrhages, cerebral brain':ab,ti | [27,797](https://www-embase-com-443.webvpn.cams.tsgvip.top/) |
| #3 | 'cerebral infarctions':ab,ti OR 'infarctions, cerebral':ab,ti OR 'infarction, cerebral':ab,ti OR 'cerebral infarct':ab,ti OR 'cerebral infarcts':ab,ti OR 'infarct, cerebral':ab,ti OR 'infarcts, cerebral':ab,ti OR 'cerebral infarction, left hemisphere':ab,ti OR 'left hemisphere, infarction, cerebral':ab,ti OR 'infarction, left hemisphere, cerebral':ab,ti OR 'left hemisphere, cerebral infarction':ab,ti OR 'cerebral, left hemisphere, infarction':ab,ti OR 'infarction, cerebral, left hemisphere':ab,ti OR 'subcortical infarction':ab,ti OR 'infarction, subcortical':ab,ti OR 'infarctions, subcortical':ab,ti OR 'subcortical infarctions':ab,ti OR 'posterior choroidal artery infarction':ab,ti OR 'anterior choroidal artery infarction':ab,ti OR 'cerebral infarction, right hemisphere':ab,ti OR 'right hemisphere, cerebral infarction':ab,ti OR 'infarction, right hemisphere, cerebral':ab,ti OR 'right hemisphere, infarction, cerebral':ab,ti OR 'cerebral, right hemisphere, infarction':ab,ti OR 'infarction, cerebral, right hemisphere':ab,ti | [9,391](https://www-embase-com-443.webvpn.cams.tsgvip.top/) |
| #2 | 'cerebrovascular accident':ab,ti OR 'cerebrovascular accidents':ab,ti OR 'cva cerebrovascular accident':ab,ti OR 'cvas cerebrovascular accident':ab,ti OR 'cerebrovascular apoplexy':ab,ti OR 'apoplexy, cerebrovascular':ab,ti OR 'vascular accident, brain':ab,ti OR 'brain vascular accident':ab,ti OR 'brain vascular accidents':ab,ti OR 'vascular accidents, brai':ab,ti OR 'cerebrovascular stroke':ab,ti OR 'cerebrovascular strokes':ab,ti OR 'stroke, cerebrovascular':ab,ti OR 'strokes, cerebrovascular':ab,ti OR 'apoplexy':ab,ti OR 'cerebral stroke':ab,ti OR 'cerebral strokes':ab,ti OR 'stroke, cerebral':ab,ti OR 'strokes, cerebral':ab,ti OR 'stroke, acute':ab,ti OR 'acute stroke':ab,ti OR 'acute strokes':ab,ti OR 'strokes, acute':ab,ti OR 'cerebrovascular accident, acute':ab,ti OR 'acute cerebrovascular accident':ab,ti OR 'acute cerebrovascular accidents':ab,ti OR 'cerebrovascular accidents, acute':ab,ti | [62,234](https://www-embase-com-443.webvpn.cams.tsgvip.top/) |
| #1 | 'stroke'/exp OR stroke | [765,503](https://www-embase-com-443.webvpn.cams.tsgvip.top/) |

**Cochrane:**

| #1 | (stroke OR Strokes OR Cerebrovascular Accident OR Cerebrovascular Accidents OR CVA Cerebrovascular Accident OR CVAs Cerebrovascular Accident OR Cerebrovascular Apoplexy OR Apoplexy, Cerebrovascular OR Vascular Accident, Brain OR Brain Vascular Accident OR Brain Vascular Accidents OR Vascular Accidents, Brai OR Cerebrovascular Stroke OR Cerebrovascular Strokes OR Stroke, Cerebrovascular OR Strokes, Cerebrovascular OR Apoplexy OR Cerebral Stroke OR Cerebral Strokes OR Stroke, Cerebral OR Strokes, Cerebral OR Stroke, Acute OR Acute Stroke OR Acute Strokes OR Strokes, Acute OR Cerebrovascular Accident, Acute OR Acute Cerebrovascular Accident OR Acute Cerebrovascular Accidents OR Cerebrovascular Accidents, Acute):ti,ab,kw | 92436 |
| --- | --- | --- |
| #2 | (Cerebral Infarction OR Cerebral Infarctions OR Cerebral Infarct OR Cerebral Infarcts OR Subcortical Infarction OR Subcortical Infarctions OR Posterior Choroidal Artery Infarction OR Anterior Choroidal Artery Infarction):ti,ab,kw | 7664 |
| #3 | (Cerebral Hemorrhage OR Cerebrum Hemorrhage OR Cerebrum Hemorrhages OR Cerebral Parenchymal Hemorrhage OR Cerebral Parenchymal Hemorrhages OR Intracerebral Hemorrhage OR Intracerebral Hemorrhages OR Cerebral Hemorrhages OR Cerebral Brain Hemorrhage OR Cerebral Brain Hemorrhages):ti,ab,kw | 8105 |
| #4 | (acupuncture OR acupunctur* OR acupoint* OR needl*):ti,ab,kw | 44880 |
| #5 | (sham acupuncture OR placebo acupuncture OR non-acupoint acupuncture OR Nonacupoints OR non-meridian points OR shallow acupuncture OR minimal acupuncture OR Superficial acupuncture OR acupoints acupuncture therapy OR non-sensitized points OR low-sensitized points OR placebo acupoint OR basic acupuncture OR non-affected meridian acupuncture):ti,ab,kw | 8044 |
| #6 | #1 OR #2 OR #3 AND #4 AND #5 AND Randomized Controlled Trial | 279 |

**Web of science:**

(((((TS=(stroke OR strokes OR Cerebrovascular Accident OR Cerebrovascular Accidents OR CVA Cerebrovascular Accident OR cmas Cerebrovascular Accident OR Cerebrovascular Apoplexy OR Vascular Accident, Brain OR Brain Vascular Accident OR Brain Vascular Accidents OR Cerebrovascular Stroke OR Cerebrovascular Strokes OR Apoplexy OR Cerebral Stroke OR Cerebral Strokes OR Acute Stroke OR Acute Strokes OR Acute Cerebrovascular Accident OR Acute Cerebrovascular Accidents )) OR TS=(Cerebral Infarction OR Cerebral Infarctions OR Cerebral Infarct OR Cerebral Infarcts OR Subcortical Infarction OR Subcortical Infarctions OR Posterior Choroidal Artery Infarction OR Anterior Choroidal Artery Infarction)) OR TS=(Cerebral Hemorrhage OR Cerebrum Hemorrhage OR Cerebrum Hemorrhages OR Cerebral Parenchymal Hemorrhage OR Cerebral Parenchymal Hemorrhages OR Intracerebral Hemorrhage OR Intracerebral Hemorrhages OR Cerebral Hemorrhages OR Cerebral Brain Hemorrhage OR Cerebral Brain Hemorrhages)) AND TS=(acupuncture OR acupunctur* OR acupoint* OR needl* )) AND TS=(sham acupuncture OR placebo acupuncture OR non-acupoint acupuncture OR nonacupoint OR non-meridian points OR shallow acupuncture OR minimal acupuncture OR Superficial acupuncture OR acupoints acupuncture therapy OR low sensitized points OR non-sensitive points OR placebo acupoint OR basic acupuncture OR non-affected meridian acupuncture)) AND TS=(Randomized Controlled Trial) and Preprint Citation Index (Exclude – Database)=208条。

**CNKI:**

发表时间 between (2000-01-01,2024-12-31) 并且 ( ( ( ( ( (旧版主题=脑卒中 或者 keyword=中英文扩展(脑卒中) 或者 title=中英文扩展(脑卒中) 或者 abstract=中英文扩展(脑卒中)) 或者 (旧版主题=中风 或者 keyword=中英文扩展(中风) 或者 title=中英文扩展(中风) 或者 abstract=中英文扩展(中风)) ) 或者 ( (旧版主题=脑梗死 或者 keyword=中英文扩展(脑梗死) 或者 title=中英文扩展(脑梗死) 或者 abstract=中英文扩展(脑梗死)) 或者 (旧版主题=脑出血 或者 keyword=中英文扩展(脑出血) 或者 title=中英文扩展(脑出血) 或者 abstract=中英文扩展(脑出血)) ) ) 并且 ( (旧版主题=针灸 或者 keyword=中英文扩展(针灸) 或者 title=中英文扩展(针灸) 或者 abstract=中英文扩展(针灸)) 并且 (旧版主题=安慰针 或者 keyword=中英文扩展(安慰针) 或者 title=中英文扩展(安慰针) 或者 abstract=中英文扩展(安慰针)) ) ) 或者 ( (旧版主题=非穴针刺 或者 keyword=中英文扩展(非穴针刺) 或者 title=中英文扩展(非穴针刺) 或者 abstract=中英文扩展(非穴针刺)) 或者 ( (旧版主题=穴位浅刺 或者 keyword=中英文扩展(穴位浅刺) 或者 title=中英文扩展(穴位浅刺) 或者 abstract=中英文扩展(穴位浅刺)) 或者 ( (旧版主题=非穴浅刺 或者 keyword=中英文扩展(非穴浅刺) 或者 title=中英文扩展(非穴浅刺) 或者 abstract=中英文扩展(非穴浅刺)) 或者 ( (旧版主题=非治疗穴 或者 keyword=中英文扩展(非治疗穴) 或者 title=中英文扩展(非治疗穴) 或者 abstract=中英文扩展(非治疗穴)) 或者 (旧版主题=假针刺 或者 keyword=中英文扩展(假针刺) 或者 title=中英文扩展(假针刺) 或者 abstract=中英文扩展(假针刺)) ) ) 并且 ( (旧版主题=随机对照 或者 keyword=中英文扩展(随机对照) 或者 title=中英文扩展(随机对照) 或者 abstract=中英文扩展(随机对照)) 不包含 (旧版主题=动物 或者 keyword=中英文扩展(动物) 或者 title=中英文扩展(动物) 或者 abstract=中英文扩展(动物)) ) ) (模糊匹配),专辑导航：全部; 数据库：文献 跨库检索=2126条

**VIP:**

(((((((题名或关键词=脑卒中 OR 题名或关键词=中风) OR 题名或关键词=脑梗死) OR 题名或关键词=脑出血) AND 题名或关键词=针灸) OR (((((题名或关键词=假针 OR 题名或关键词=安慰针) OR 题名或关键词=非穴针刺) OR 题名或关键词=穴位浅刺) OR 题名或关键词=非穴浅刺) OR 题名或关键词=非治疗穴)) AND 题名或关键词=随机对照) AND ( NOT 题名或关键词=动物)) AND (years:[2000 TO 2024])=101条

Wan-fang:

(题名或关键词:("脑卒中 or 中风 or 脑梗死 or 脑出血") and 题名或关键词:("针刺") or 题名或关键词:(假针 or 安慰针 or 非穴针刺or 穴位浅刺 or 非穴浅刺or 非治疗穴) and 题名或关键词:("随机对照") not 题名或关键词:("动物")) and Date:2000-2024=126条

# Supplementary Table 1. Characteristics of patients and treatment time details included in studies.

| **Source** | **Patients, No**  **(IG/CG)** | **Patients age of IG**  **/y**  **(Mean±SD)** | **Patients age of CG**  **/y**  **(Mean±SD)** | **Treatment frequency**  **/week** | **Treatment duration** | **Follow-up time** |
| --- | --- | --- | --- | --- | --- | --- |
| Shen et al. 2012 | 144/143 | 60.24±9.30 | 61.41±9.82 | 7times | 4wks | 6mo |
| Xia et al. 2016 | 61/55 | 67.00±9.00 | 66.00±10.00 | 5times | 6wks | 3mo |
| Li et al. 2014 | 121/117 | 63.20±10.49 | 64.21±10.19 | 5times | 4wks | 12wks |
| You et al. 2020 | 34/33 | NR | NR | 6times | 4wks | 8wks |
| Li et al. 2011 | 23/20 | 56.70±14.40 | 59.40±12.10 | 5times | 6wks | NR |
| Lee et al. 2009 | 27/25 | 66.70±11.00 | 66.00±9.60 | Only once | 3days | NR |
| Zheng et al. 2011 | 41/37 | 66.98±10.19 | 65.05±9.92 | 5times | 4wks | NR |
| Liao et al. 2017 | 28/20 | 62.29±12.33 | 55.45±15.22 | 3times | 8wks | 24wks |
| Tsai et al. 2024 | 18/19 | 69.90±12.00 | 69.10±9.10 | 2times | 4wks | NR |
| Li et al. 2023 | 35/37 | 58.20±12.70 | 60.60±11.2 | 7times | 2wks | NR |
| Li et al. 2024 | 115/116 | 61.30±7.10 | 59.60±7.70 | 5times | 6wks | 6mo |
| Liu et al. 2013 | 33/33 | 39.00±12.00 | 37.00±11.00 | 7times | 10days | NR |
| Feixiang,Ma-2024 | 72/71 | 54.65±7.07 | 56.10±5.93 | 5times | 6wks | 10wks |
| Wang et al. 2023 | 30/29 | 67.00(53.00,74.00)^a^ | 62.00(52.00,71.00) ^a^ | 5times | 3wks | NR |
| Liu et al. 2021 | 30/30 | 55.42±3.97 | 56.12±3.77 | 5times | 6wks | NR |
| Xiong et al. 2020 | 35/35 | 63.00±7.23 | 65.30±8.52 | 6times | 8wks | NR |
| Qian et al. 2015 | 32/33 | 67.59±11.02 | 67.74±8.61 | 7times | 6wks | 3mo |
| Li ZT et al. 2023 | 34/34 | 69.00±8.00 | 67.00±9.00 | 3times | 4wks | NR |
| Li T et al. 2023 | 30/30 | 66.40±9.00 | 66.60±6.30 | 5times | 4wks | NR |
| Zheng et al. 2023 | 60/60 | 60.61±6.75 | 59.72±7.32 | 5times | 6wks | NR |
| Li MC et al. 2024 | 26/28 | 65.00(51.5,74.5) ^a^ | 64.50(50.00,72.00) ^a^ | 5times | 3wks | NR |
| Cao et al. 2022 | 72/72 | 65.20±7.10 | 63.60±8.25 | 3times | 4wks | 8wks |
| Cai et al. 2022 | 33/32 | 67.8±10.91 | 66.70±11.42 | 3times | 4wks | 8wks |
| Zhang et al. 2024 | 34/33 | NR | NR | 5times | 3wks | NR |

Abbreviations: CG, Control Group; IG, Intervention Group; NR, Not Reported.

^a^, mean(P25,P75).

# Supplementary Table 2. Details of acupuncture treatment in intervention group vs control group.

| **Source** | **Intervention group(IG)** | | | |  | **Control group(CG)** | | | | | | | | **Retention time** |
| --- | --- | --- | --- | --- | --- | --- | --- | --- | --- | --- | --- | --- | --- | --- |
|  | **Treatment** | **Manually manipulate** | **De qi** | **Eacu** |  | **Treatment** | **Location** | **Acupoints, No.** | **Needling instrument** | **Depth** | **Manually manipulate** | **De qi** | **Eacu** |  |
| Shen et al. 2012 | AT | Yes | Yes | None |  | SAT | Nacu-X(1cm or the midpoint of two meridians) | Same as the IG | CDSAN | Same as the IG | Yes | Yes | None | NR |
| Xia et al. 2016 | AT | Yes | Yes | None |  | SAT | Nacu-X(5cm) | Same as the IG | CDSAN | SPUS(1-4mm) | None | None | None | 30min |
| Li et al. 2014 | AT | Yes | Yes | None |  | SAT | Nacu-X(0.1cun) | Same as the IG | CDSAN | 5mm | None | None | None | 30min |
| You et al. 2020 | AT | None | None | None |  | SAT | Same as the IG | Same as the IG | BNNI | NP | None | None | None | 30min |
| Li et al. 2011 | AT, PP | Yes | Yes | None |  | SAT, Drug | Nacu-X(0.5cm) | NR | CDSAN | SPUS(1-4mm) | None | NR | None | 30min |
| Lee et al. 2009 | AT | NR | NR | None |  | SAT | Same as the IG | Same as the IG | BNNI | NP | None | None | None | 3days |
| Zheng et al. 2011 | AT | Yes | Yes | None |  | SAT | Same as the IG | Same as the IG | RSA | NP | None | None | None | 30min |
| Liao et al. 2017 | AT | NR | Yes | None |  | SAT | Nacu-X(1cm) | Same as the IG | CDSAN | SPUS(5mm) | None | None | None | 20min |
| Tsai et al. 2024 | AT | Yes | Yes | None |  | SAT | Nacu-X(2cm) | Same as the IG | CDSAN | SPUS | None | None | None | 20min |
| Li et al. 2023 | AT | Yes | Yes | None |  | SAT | Same as the IG | Same as the IG | CDSAN, sham Eacu | NP | None | None | None | 30min |
| Boxuan, Li-2024 | AT | Yes | Yes | None |  | SAT | Nacu-X(1cun) | Same as the IG | CDSAN | SPUS(0.2cun) | None | None | None | 30min |
| Liu et al. 2013 | Eacu | Yes | Yes | 30-40Hz, continuous wave |  | Sham Eacu | Nacu-X(1cm) | Same as the IG | Sham Eacu | NP | None | None | Sham Eacu | 20min |
| Ma et al. 2024 | Eacu, PP | Yes | Yes | 2/15Hz |  | Sham Eacu, Drug | Nacu-X(0.5-1cm) | Same as the IG | CDSAN | SPUS(3-5mm) | None | NR | Sham Eacu | 30min |
| Wang et al. 2023 | AT | None | Yes | None |  | SAT | Same as the IG | Same as the IG | BNNI | NP | None | None | None | 30min |
| Liu et al. 2021 | AT, PP | None | Yes | None |  | SAT, Drug | Nacu-X(0.5cm) | Same as the IG | CDSAN | SPUS(1-4mm) | None | NR | None | 30min |
| Xiong et al. 2020 | AT | NR | NR | None |  | SAT | Nacu-X(the midpoint of two acupoints) | 1 | RSA | NP | None | None | None | 3-4hours |
| Qian et al. 2015 | AT, PP | Yes | Yes | None |  | SAT, Drug | Nacu-X(0.5cm) | Same as the IG | CDSAN | SPUS(1-4mm) | None | NR | None | 30min |
| Li ZT et al. 2023 | AT | Yes | NR | None |  | SAT | Nacu-RR | 4 | CDSAN | SPUS(3-5mm) | None | None | None | 30min |
| Li T et al. 2023 | Eacu | Yes | Yes | 5-8Hz, continuous wave |  | Mini-eacu | Nacu-X(1cm) | Same as the IG | CDSAN | 15mm | None | None | Mini-ES | 30min |
| Zheng et al. 2023 | Eacu | Yes | Yes | 2Hz, continuous wave |  | Mini-eacu | Same as the IG | Same as the IG | BNNI | NP | None | None | Mini-ES | 30min |
| Li MC et al. 2024 | AT | None | Yes | None |  | SAT | Same as the IG | Same as the IG | BNNI | NP | None | None | None | 30min |
| Cao et al. 2022 | AT | None | Yes | None |  | SAT | Same as the IG | Same as the IG | CDSAN | NP | None | NR | None | 20min |
| Cai et al. 2022 | Eacu | Yes | Yes | 2/100Hz |  | Sham Eacu | Same as the IG | Same as the IG | BNNI | NP | None | None | Sham Eacu | 30min |
| Zhang et al. 2024 | Eacu | None | NR | 2Hz |  | SAT, Mini-eacu | Nacu-RR | Same as the IG | BNNI | NP | None | None | Mini-ES | 20min |

Abbreviations: AT, Acupuncture Therapy; BNNI, Blunt needle not inserted; CDSAN, Conventional disposable sterile acupuncture needles; Eacu, Electroacupuncture; ES, Electrical Stimulation; Nacu-RR, Non-acupoints Selected at Remote Regions; Nacu-X, Non-acupoints Selected at X cm Lateral to the Acupoint; NP, Not penetrated; NR, Not reported; PP, Pharmacological Placebo; RSA, Retractable Sham Acupuncture; SAT, Sham Acupuncture Therapy; SPUS, Shallow puncture under the skin.

# Supplementary Table 3. Study risk of bias.

| **Study ID** | **Randomization process** | **Deviations from the intended interventions** | **Missing outcome data** | **Measurement of the outcome** | **Selection of the reported result** | **Overall** |
| --- | --- | --- | --- | --- | --- | --- |
| Shen et al. 2012 |  |  |  |  |  |  |
| Xia et al. 2016 |  |  |  |  |  |  |
| Li et al. 2014 |  |  |  |  |  |  |
| You et al. 2020 |  |  |  |  |  |  |
| Li et al. 2011 |  |  |  |  |  |  |
| Lee et al. 2009 |  |  |  |  |  |  |
| Zheng et al. 2011 |  |  |  |  |  |  |
| Liao et al. 2017 |  |  |  |  |  |  |
| Tsai et al. 2024 |  |  |  |  |  |  |
| Li et al. 2023 |  |  |  |  |  |  |
| Li et al. 2024 |  |  |  |  |  |  |
| Liu et al. 2013 |  |  |  |  |  |  |
| Ma et al. 2024 |  |  |  |  |  |  |
| Wang et al. 2023 |  |  |  |  |  |  |
| Liu et al. 2021 |  |  |  |  |  |  |
| Xiong et al. 2020 |  |  |  |  |  |  |
| Qian et al. 2015 |  |  |  |  |  |  |
| Li ZT et al. 2023 |  |  |  |  |  |  |
| Li T et al. 2023 |  |  |  |  |  |  |
| Zheng et al. 2023 |  |  |  |  |  |  |
| Li MC et al. 2024 |  |  |  |  |  |  |
| Cao et al. 2022 |  |  |  |  |  |  |
| Cai et al. 2022 |  |  |  |  |  |  |
| Zhang et al. 2024 |  |  |  |  |  |  |
| Low risk | Some concerns | | | High risk | | |

# Supplementary Table 4. STRICTA checklist for the included studies.

| **Author (Year)** | **1 Acupuncture rationale** | | | **2 Details of needling** | | | | | | | **3 Treatment regimen** | | **4 Complementary interventions** | | **5 Practitioner background** | **6 Control or comparator interventions** | |
| --- | --- | --- | --- | --- | --- | --- | --- | --- | --- | --- | --- | --- | --- | --- | --- | --- | --- |
|  | **1a** | **1b** | **1c** | **2a** | **2b** | **2c** | **2d** | **2e** | **2f** | **2g** | **3a** | **3b** | **4a** | **4b** | **5** | **6a** | **6b** |
| **Shen et al. 2012** | Y | Y | Y | N | Y | Y | Y | Y | N | Y | Y | Y | Y | Y | N | N | N |
| **Xia et al. 2016** | Y | Y | Y | N | Y | Y | Y | Y | Y | Y | Y | Y | Y | Y | N | N | N |
| **Li et al. 2014** | Y | Y | N | Y | Y | Y | Y | Y | Y | Y | Y | Y | N | Y | Y | N | N |
| **You et al. 2020** | Y | Y | Y | N | Y | N | Y | N | Y | Y | Y | Y | Y | Y | Y | Y | Y |
| **Li et al. 2011** | Y | Y | N | N | Y | Y | Y | Y | Y | Y | Y | Y | Y | Y | N | N | Y |
| **Lee et al. 2009** | Y | Y | N | Y | Y | N | N | N | Y | Y | N | N | N | Y | N | N | N |
| **Zheng et al. 2011** | Y | Y | Y | N | Y | N | Y | Y | Y | Y | Y | Y | N | Y | N | N | N |
| **Liao et al. 2017** | Y | Y | Y | N | Y | Y | Y | Y | Y | Y | Y | Y | N | Y | Y | N | Y |
| **Tsai et al. 2024** | Y | Y | N | Y | Y | Y | Y | Y | Y | Y | Y | Y | N | Y | Y | Y | Y |
| **Li et al. 2023** | Y | Y | N | Y | Y | Y | Y | Y | Y | Y | Y | Y | N | Y | Y | Y | N |
| **Li et al. 2024** | Y | Y | N | Y | Y | Y | Y | Y | Y | Y | Y | Y | Y | Y | N | Y | Y |
| **Liu et al. 2013** | Y | Y | Y | Y | Y | Y | Y | Y | Y | Y | Y | Y | N | Y | Y | Y | Y |
| **Ma et al. 2024** | Y | Y | N | Y | Y | Y | Y | Y | Y | Y | Y | Y | N | Y | Y | N | Y |
| **Wang et al. 2023** | Y | Y | Y | N | Y | Y | Y | Y | Y | Y | Y | Y | N | Y | Y | Y | N |
| **Liu et al. 2021** | Y | Y | N | Y | Y | Y | Y | Y | Y | Y | Y | Y | N | Y | N | N | Y |
| **Xiong et al. 2020** | Y | Y | N | Y | Y | Y | N | Y | Y | Y | Y | Y | N | Y | N | N | Y |
| **Qian et al. 2015** | Y | Y | N | Y | Y | Y | Y | Y | Y | Y | Y | Y | Y | Y | N | N | N |
| **Li ZT et al. 2023** | Y | Y | N | Y | Y | Y | N | Y | Y | N | Y | Y | N | Y | Y | Y | Y |
| **Li T et al. 2023** | Y | Y | N | Y | Y | Y | Y | Y | Y | Y | Y | Y | N | Y | N | Y | Y |
| **Zheng et al. 2023** | Y | Y | N | Y | Y | Y | Y | Y | Y | Y | Y | Y | N | Y | N | N | N |
| **Li MC et al. 2024** | Y | Y | N | Y | Y | Y | Y | N | Y | Y | Y | Y | N | Y | N | Y | Y |
| **Cao et al. 2022** | Y | Y | N | Y | Y | Y | Y | Y | Y | Y | Y | Y | N | Y | N | N | Y |
| **Cai et al. 2022** | Y | Y | N | N | Y | Y | Y | Y | Y | N | Y | Y | N | Y | N | Y | N |
| **Zhang et al. 2024** | Y | Y | N | Y | Y | Y | N | N | Y | Y | Y | Y | N | Y | Y | Y | N |

Note:

1a) Style of acupuncture (e.g. Traditional Chinese Medicine, Japanese, Korean, Western medical, etc);

1b) Reasoning for treatment provided, literature sources, and/or consensus methods, with references where appropriate;

1c) Extent to which treatment was varied;

2a) Number of needle insertions per subject per session (mean and range where relevant) ;

2b) Names (or location if no standard name) of points used (uni/bilateral) ;

2c) Depth of insertion, based on a specified unit of measurement;

2d) Response sought (e.g. de qi or muscle twitch response);

2e) Needle stimulation (e.g. manual，electrical) ;

2f) Needle retention time;

2g) Needle type (diameter, length, and manufacturer) ;

3a) Number of treatment sessions;

3b) Frequency and duration of treatment sessions;

4a) Details of other interventions administered to the acupuncture group (e.g. moxibustion, cupping, herbs, exercises) ;

4b) Setting and context of treatment, including instructions to practitioners, and information and explanations to patients;

5) Description of participating acupuncturists (qualification or professional affiliation, other relevant experience) ;

6a) Rationale for the control or comparator in the context of the research question, with sources that justify this choice;

6b) Precise description of the control or comparator. If sham acupuncture or any other type of acupuncture-like control is used, provide details as for Items 1 to 3 above.

N, not adequately reported; Y, adequately reported; Ya, reported but did not mention unilateral or bilateral; STRICTA, Standards for Reporting Interventions in Clinical Trials of Acupuncture.

# Supplementary Table 5. Summary of findings for the main comparison.

| **Outcomes** | **No of patients**  **IG/CG(Studies)** | **Effect** | | **Quality (GRADE)** | **Importance** | **Comments** |
| --- | --- | --- | --- | --- | --- | --- |
|  |  | **Relative**  **(95% CI)** | **Absolute** |  |  |  |
| Barthel Index | 233/221  (5 RCTs) | - | 0.51 to 4.66 higher  (MD 2.58 higher) | ÅÅOO  Low^1,2^ | Critical | Most studies were at high or unclear risk of bias. All studies were conducted in China. |
| National Institute of Health Stroke Scale | 488/475  (7 RCTs) | - | -1.94 to -0.26 lower  (MD -1.10 lower) | ÅÅOO  Low^1,3^ | Critical | Substantial heterogeneity in results. Most studies were at high or unclear risk of bias. All studies were conducted in China. |
| Stroke Specific Quality of Life Scale | 380/376  (3 RCTs) | - | 6.02 to 21.80 higher  (MD 13.91 higher) | ÅÅÅO  Moderate^1,4^ | Critical | All studies were conducted in China. |
| Hamilton depression scale | 187/174  (5 RCTs) | - | -1.11 to -0.03 lower  (SMD -0.54 lower) | ÅÅOO  Low ^1,4,5^ | Important | Substantial heterogeneity in results. Most studies were at high or unclear risk of bias. All studies were conducted in China. |

CG, Control Group; CI: Confidence interval; IG, Intervention Group; MD, Mean difference; SMD, Standardized mean difference.

1 Downgraded by two levels for very serious risk of bias (most of them did not blind acupuncturists, and most of them were at risk of attrition bias).

2 Downgraded by one level for serious imprecision (passing through line of no effect).

3 Downgraded by one level for serious inconsistency (due to substantial heterogeneity, I^2^ = 50%).

4 Although the results showed substantial heterogeneity(I^2^﹥50%), it was confirmed that the source of heterogeneity may be related to the sham acupuncture methods.

5 Downgraded by one level for serious imprecision (n＜400).

# Supplementary Figure 1. Risk of bias graph: each risk of bias item presented as percentages across all included studies.


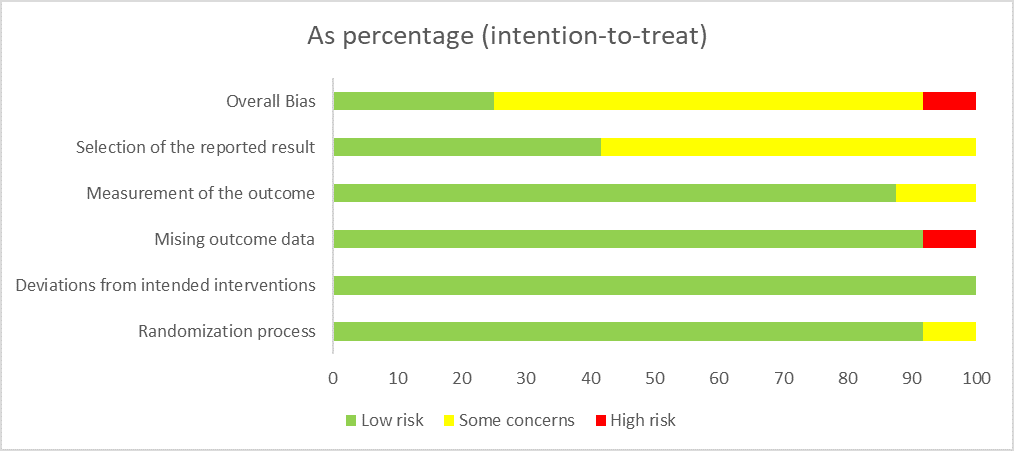


# Supplementary Figure 2. Comparison of the effects of different sham acupuncture.


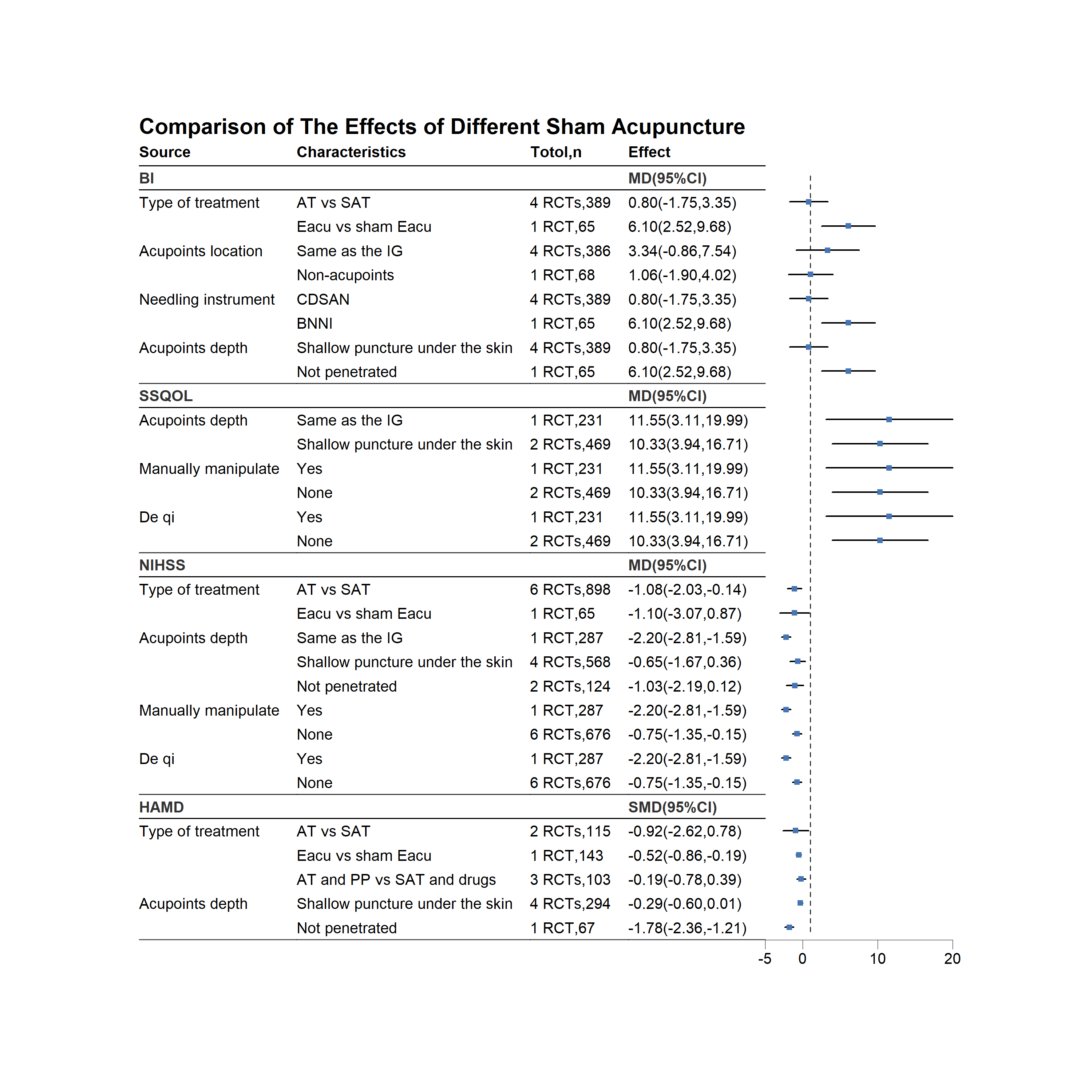


# Supplementary Figure 3. Summary of the adverse.


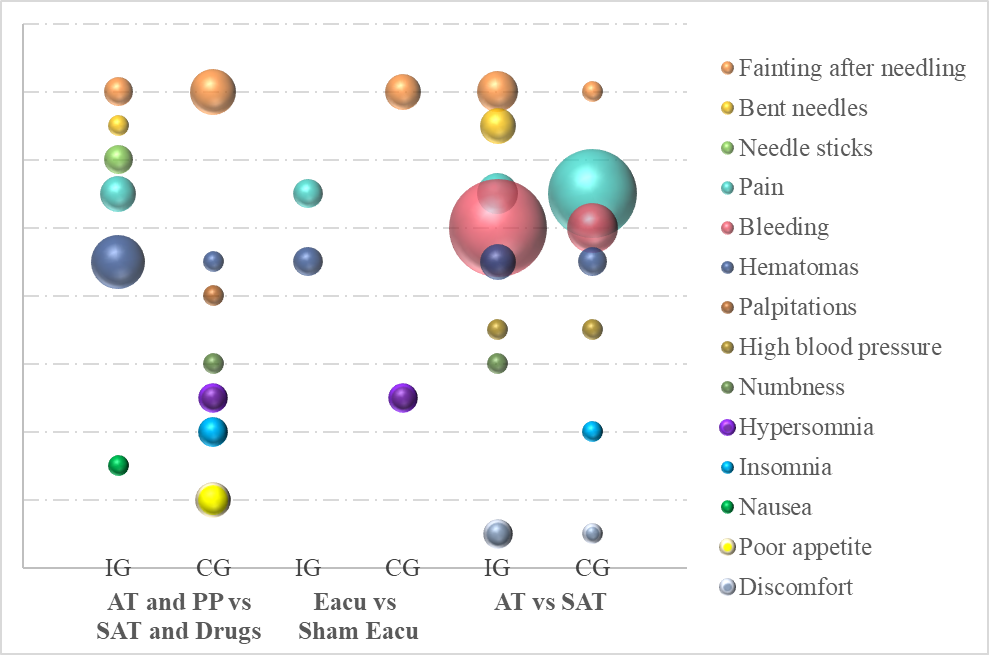

Supplement: Supplementary file 1 [file Table_1.docx]
